# Therapy Intensity Level Scale for Traumatic Brain Injury: Clinimetric Assessment on Neuro-Monitored Patients Across 52 European Intensive Care Units

**DOI:** 10.1089/neu.2023.0377

**Published:** 2024-04-04

**Authors:** Shubhayu Bhattacharyay, Erta Beqiri, Patrick Zuercher, Lindsay Wilson, Ewout W. Steyerberg, David W. Nelson, Andrew I. R. Maas, David K. Menon, Ari Ercole

**Affiliations:** ^1^ Division of Anaesthesia, Division of Neurosurgery, University of Cambridge, Cambridge, United Kingdom.; ^2^ Department of Clinical Neurosciences, Division of Neurosurgery, University of Cambridge, Cambridge, United Kingdom.; ^3^ Brain Physics Laboratory, Division of Neurosurgery, University of Cambridge, Cambridge, United Kingdom.; ^4^ Department of Intensive Care Medicine, Inselspital, Bern University Hospital, University of Bern, Switzerland.; ^5^ Division of Psychology, University of Stirling, Stirling, United Kingdom.; ^6^ Department of Biomedical Data Sciences, Leiden University Medical Center, Leiden, the Netherlands.; ^7^ Department of Physiology and Pharmacology, Section for Perioperative Medicine and Intensive Care, Karolinska Institutet, Stockholm, Sweden.; ^8^ Department of Neurosurgery, Antwerp University Hospital, Edegem, Belgium.; ^9^ Department of Translational Neuroscience, Faculty of Medicine and Health Science, University of Antwerp, Antwerp, Belgium.; ^10^ Cambridge Center for Artificial Intelligence in Medicine, Cambridge, United Kingdom.

**Keywords:** clinimetrics, intensive care unit, intracranial pressure, Therapy Intensity Level, traumatic brain injury, validation

## Abstract

Intracranial pressure (ICP) data from traumatic brain injury (TBI) patients in the intensive care unit (ICU) cannot be interpreted appropriately without accounting for the effect of administered therapy intensity level (TIL) on ICP. A 15-point scale was originally proposed in 1987 to quantify the hourly intensity of ICP-targeted treatment. This scale was subsequently modified—through expert consensus—during the development of TBI Common Data Elements to address statistical limitations and improve usability. The latest 38-point scale (hereafter referred to as TIL) permits integrated scoring for a 24-h period and has a five-category, condensed version (TIL^(Basic)^) based on qualitative assessment. Here, we perform a total- and component-score analysis of TIL and TIL^(Basic)^ to: 1) validate the scales across the wide variation in contemporary ICP management; 2) compare their performance against that of predecessors; and 3) derive guidelines for proper scale use. From the observational Collaborative European NeuroTrauma Effectiveness Research in TBI (CENTER-TBI) study, we extract clinical data from a prospective cohort of ICP-monitored TBI patients (*n* = 873) from 52 ICUs across 19 countries. We calculate daily TIL and TIL^(Basic)^ scores (TIL_24_ and TIL^(Basic)^_24_, respectively) from each patient's first week of ICU stay. We also calculate summary TIL and TIL^(Basic)^ scores by taking the first-week maximum (TIL_max_ and TIL^(Basic)^_max_) and first-week median (TIL_median_ and TIL^(Basic)^_median_) of TIL_24_ and TIL^(Basic)^_24_ scores for each patient. We find that, across all measures of construct and criterion validity, the latest TIL scale performs significantly greater than or similarly to all alternative scales (including TIL^(Basic)^) and integrates the widest range of modern ICP treatments. TIL_median_ outperforms both TIL_max_ and summarized ICP values in detecting refractory intracranial hypertension (RICH) during ICU stay. The RICH detection thresholds which maximize the sum of sensitivity and specificity are TIL_median_ ≥ 7.5 and TIL_max_ ≥ 14. The TIL_24_ threshold which maximizes the sum of sensitivity and specificity in the detection of surgical ICP control is TIL_24_ ≥ 9. The median scores of each TIL component therapy over increasing TIL_24_ reflect a credible staircase approach to treatment intensity escalation, from head positioning to surgical ICP control, as well as considerable variability in the use of cerebrospinal fluid drainage and decompressive craniectomy. Since TIL^(Basic)^_max_ suffers from a strong statistical ceiling effect and only covers 17% (95% confidence interval [CI]: 16–18%) of the information in TIL_max_, TIL^(Basic)^ should not be used instead of TIL for rating maximum treatment intensity. TIL^(Basic)^_24_ and TIL^(Basic)^_median_ can be suitable replacements for TIL_24_ and TIL_median_, respectively (with up to 33% [95% CI: 31–35%] information coverage) when full TIL assessment is infeasible. Accordingly, we derive numerical ranges for categorising TIL_24_ scores into TIL^(Basic)^_24_ scores. In conclusion, our results validate TIL across a spectrum of ICP management and monitoring approaches. TIL is a more sensitive surrogate for pathophysiology than ICP and thus can be considered an intermediate outcome after TBI.

## Introduction

Elevated intracranial pressure (ICP) following traumatic brain injury (TBI) may impede the potential recovery of injured brain tissue and damage initially unaffected brain regions.^1^ Therefore, for TBI patients admitted to the intensive care unit (ICU), clinicians often monitor ICP and apply a wide range of ICP-reducing treatments.^2^ The selective use of these treatments typically follows a staircase approach, in which therapeutic intensity—defined by the risk and complexity of each treatment—is incrementally escalated until adequate ICP control is achieved.^3-5^ Thus, therapeutic intensity must be considered when interpreting ICP. Even if two TBI patients have comparable ICP values, a difference in the intensity of their ICP-directed therapies likely indicates a difference in pathophysiological severity.

Several versions of the Therapy Intensity Level (TIL) scale have been developed to rate and compare the overall intensity of ICP management amongst TBI patients. TIL scales assign a relative intensity score to each ICP-targeting therapy and return either the sum or the maximum value of the scores of simultaneously applied therapies. In 1987, Maset and colleagues produced the original, 15-point TIL scale (TIL^(1987)^) to be assessed once every 4 h.^6^ In 2006, Shore and colleagues published the 38-point Pediatric Intensity Level of Therapy (PILOT) scale,^7^ revising TIL^(1987)^ to: 1) represent updated pediatric TBI management practices; 2) have a more practical, daily assessment frequency; and 3) resolve a statistical ceiling effect. In 2011, the inter-agency TBI Common Data Elements (CDE) scheme developed the most recent, 38-point TIL scale (hereafter referred to as TIL) as well as a condensed, five-category TIL^(Basic)^ scale through expert consensus.^8^ The TIL scale revised PILOT to integrate additional ICP-directed therapies and to be applicable to adult TBI management. Moreover, TIL^(Basic)^ was proposed as a simple, categorical measure to use when full TIL assessment would be infeasible. Since Zuercher and colleagues reported the validity and reliability of TIL in a two-center cohort (*n* = 31) in 2016,^9^ the scale has become a popular research metric for quantifying ICP treatment intensity.^10–13^

However, several critical questions regarding TIL remain unanswered. It is uncertain whether the validity of TIL, reported in a relatively small population, can be generalized across the wide variation of ICP management, monitoring, and data acquisition (i.e., intermittent chart recording or high-resolution storage)^14^ strategies practiced in contemporary intensive care.^11,12,15,16^ Further, the scoring configuration of TIL has never been tested against alternatives (e.g., TIL^(1987)^ and PILOT), and the relative contribution of TIL's component therapies towards the total score is unknown. It is unclear how TIL^(Basic)^ numerically relates to TIL and if the former captures the essential information of the latter. In this work, we aimed to answer these questions by performing a comprehensive assessment of TIL on a large, contemporary population of ICP-monitored TBI patients across European ICUs.

## Methods

### Therapy Intensity Level (TIL) and alternative scales

TIL refers to the 38-point scale developed by the CDE scheme for TBI.^8^ The domain or construct (i.e., targeted concept of a scale) of TIL is the therapeutic intensity of ICP management. The TIL scale has 12 items, each representing a distinct ICP-targeting treatment from one of eight modalities, as defined in Table 1. TIL was developed by an international expert panel, which discussed: 1) the relevant ICP-treatment modalities of modern intensive care; 2) the relative risk and efficacy of individual therapies to derive scores; and 3) practical and statistical limitations of previous TIL scores.^8^ In this way, TIL is a formative measurement model in which the construct (i.e., ICP treatment intensity) is not unidimensional but rather defined by the combination of items (i.e., ICP-targeting treatments).^17^ TIL was shown to have high inter-rater and intra-rater reliability by Zuercher and colleagues.^9^ If a decompressive craniectomy was performed as a last resort for refractory intracranial hypertension, its score was included in the day of the operation and in every subsequent day of ICU stay. TIL scores can be calculated as frequently as clinically desired. For our analysis, we calculated the following TIL scores from the first 7 days of ICU stay:

**Table 1.** Scoring Configurations for TIL and Alternative Scales

ICP-treatment modality	Item	TIL		uwTIL		TIL^(Basic)^^a^	PILOT^b^		TIL^(1987)^^b^	
	Sub-item	Score	Max	Score	Max	Score^a^	Score	Max	Score	Max
Positioning	Head elevation for ICP control or nursed flat (180°) for CPP management	1	1	1	1	1	–	–	–	–
Sedation and neuromuscular blockade	Sedation		5		3			5		4
	Low dose sedation (as required for mechanical ventilation).	1		1		1	1		1	
	Higher dose sedation for ICP control (but not aiming for burst suppression).	2		2		2	1		1	
	High dose propofol or barbiturates for ICP control (metabolic suppression).	5		3		4	5		4	
	Neuromuscular blockade (paralysis).	3	3	1	1	–	2	2	1	1
CSF drainage	CSF drainage volume		3		2			5		2
	Low (<120 mL/24h)	2		1		2	4		1	
	High (≥120 mL/24h)	3		2		3	5		2	
CPP management	Fluid loading for maintenance of cerebral perfusion.	1	1	1	1	2	–	–	–	–
	Vasopressor therapy required for management of cerebral perfusion.	1	1	1	1	2	2	2	–	–
Ventilatory management	Hypocapnia for ICP control (P_a_CO_2_ [mm Hg])		4		3			4		2
	Mild (35 ≤ P_a_CO_2_<40)	1		1		2	1		1	
	Moderate (30 ≤ P_a_CO_2_<35)	2		2		3	2		1	
	Intensive (P_a_CO_2_<30)	4		3		4	4		2	
Hyperosmolar therapy	Mannitol administration		3		2			3		6
	≤2g/kg/24h	2		1		2	2		3	
	>2g/kg/24h	3		2		3	3		6	
	Hypertonic saline administration		3		2			3	–	–
	≤0.3g/kg/24h	2		1		2	3			
	>0.3g/kg/24h	3		2		3	3			
Temperature control	Temperature control (T [°C])		5		3			5	–	–
	Fever control (>38 or spontaneous <34.5).	1		1			1			
	Cooling for ICP control (≥35)	2		2		3	3			
	Hypothermia (<35).	5		3		4	5			
Surgery for intracranial hypertension	Intracranial operation for progressive mass lesion, NOT scheduled on admission.	4	4	1	1	4	4	4	–	–
	Decompressive craniectomy.	5	5	1	1	4	5	5	–	–
Maximum total possible score			38		21	4		38		15

The TIL scale was developed by Maas and colleagues.^8^ For each calendar day, the highest score for each item was summed to derive the TIL score.

^a^ TIL^(Basic)^ is the maximum score (up to 4) among all administered sub-items over the calendar day. If no sub-items are administered on a given day, TIL^(Basic)^ = 0.

^b^ PILOT scale^7^ and TIL^(1987)^ scale^6^ scoring configurations have been adapted with minor adjustments to fit the items of TIL with a daily assessment frequency.

CPP, cerebral perfusion pressure; CSF, cerebrospinal fluid; ICP, intracranial pressure; P_a_CO_2_, partial pressure of carbon dioxide in arterial blood; PILOT, Pediatric Intensity Level of Therapy scale^7^; T, body temperature in degrees Celsius; TIL, Therapy Intensity Level scale^8,9^; TIL^(1987)^, original Therapy Intensity Level scale published in 1987^6^; TIL^(Basic)^, condensed TIL scale^8^; uwTIL, unweighted TIL scale in which sub-item scores are replaced by the ascending rank index within the item.

- TIL_24_, the daily TIL score based on the sum of the highest scores per item per calendar day,
- TIL_max_, the maximum TIL_24_ over the first week of a patient's ICU stay,
- TIL_median_, the median TIL_24_ over the first week of a patient's ICU stay.

We also calculated scores from four other therapeutic intensity scales to compare with TIL scores. The 21-point, unweighted TIL (uwTIL) scale replaces each sub-item score in TIL with its ascending rank index (i.e., 1, 2, 3, …) within each item (Table 1). The five-category TIL^(Basic)^ was also developed by the CDE scheme for TBI and takes the maximum score, from zero (i.e., no ICP-related intervention) to four, amongst all included sub-items over the calendar day.^8^ We adapted the 38-point PILOT^7^ and 15-point TIL^(1987)^ scales^6^ with minor adjustments to fit the items of TIL with a daily assessment frequency. PILOT also was shown to have high inter-rater and intra-rater reliability by Shore and colleagues.^7^ For the four alternative scales, daily (i.e., uwTIL_24_, TIL^(Basic)^_24_, PILOT_24_, and TIL^(1987)^_24_), maximum (i.e., uwTIL_max_, TIL^(Basic)^_max_, PILOT_max_, and TIL^(1987)^_max_), and median (i.e., uwTIL_median_, TIL^(Basic)^_median_, PILOT_median_, and TIL^(1987)^_median_) scores were calculated in the same way as TIL_24_, TIL_max_, and TIL_median_, respectively.

### Study design and populations

Our study population was prospectively recruited for the Collaborative European NeuroTrauma Effectiveness Research in Traumatic Brain Injury (CENTER-TBI) core and high-resolution studies. CENTER-TBI is a longitudinal, observational cohort study (NCT02210221) involving 65 medical centers across 18 European countries and Israel. Patients were recruited between December 19, 2014, and December 17, 2017, if they met the following criteria: 1) presentation within 24 h of a TBI; 2) clinical indication for a CT scan; and 3) no severe pre-existing neurological disorder. In accordance with relevant laws of the European Union and the local country, ethical approval was obtained for each site, and written informed consent by the patient or legal representative was documented electronically. The list of sites, ethical committees, approval numbers, and approval dates can be found online at https://www.center-tbi.eu/project/ethical-approval. The project objectives and design of CENTER-TBI have been described in detail previously.^18,19^

**FIG. 1.**
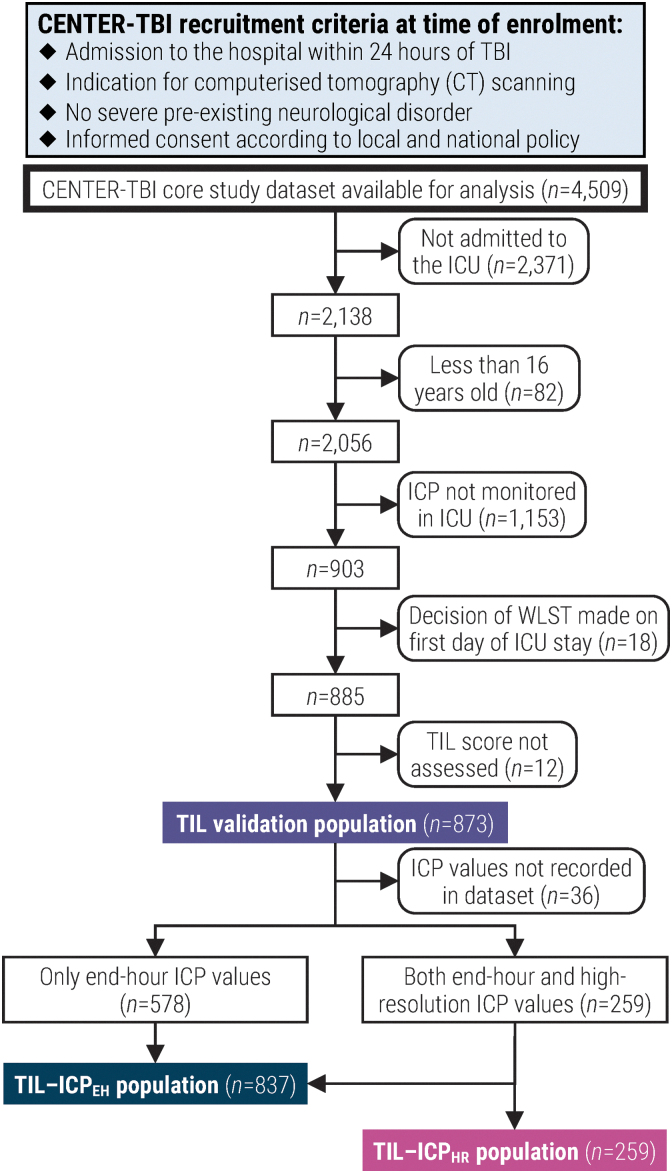
Flow diagram for patient enrollment and validation population assignment. CENTER-TBI, Collaborative European NeuroTrauma Effectiveness Research in TBI; ICP, intracranial pressure; ICP_EH_, end-hour ICP; ICP_HR_, high-resolution ICP; ICU, intensive care unit; TBI, traumatic brain injury; TIL, Therapy Intensity Level scale^8,9^; WLST, withdrawal of life-sustaining therapies.

In this work, we applied the following inclusion criteria in addition to those of CENTER-TBI (Fig. 1): 1) primary admission to the ICU; 2) at least 16 years old at ICU admission; 3) invasive ICP monitoring; 4) no decision to withdraw life-sustaining therapies (WLST) on the first day of ICU stay; and 5) daily assessment of TIL.

For our sub-studies evaluating the association between TIL and ICP-derived values, we created two sub-populations based on the type of ICP values available. Patients with end-hour ICP (ICP_EH_) values, which were recorded by clinicians at the end of every other hour, constituted the TIL-ICP_EH_ sub-population. Patients with high-resolution ICP values (ICP_HR_), which were automatically stored with monitoring software, constituted the TIL-ICP_HR_ sub-population. All patients in the TIL-ICP_HR_ sub-population were also members of the TIL-ICP_EH_ sub-population (Fig. 1).

### Data collection

Data for the CENTER-TBI study was collected through the QuesGen electronic case report form system (QuesGen Systems Inc, Burlingame, CA, USA) hosted on the International Neuroinformatics Coordinating Facility (INCF) platform (INCF, Stockholm, Sweden). All data for the validation populations, except high-resolution signals, were extracted from the CENTER-TBI core study^19^ (v3.0, ICU stratum) using Opal database software.^20^

### ICP management data for TIL calculation

Since TIL_24_ was found to be a reliable summary of hourly TIL,^9^ clinical data pertinent to the component items of TIL (i.e., ICP-guided treatments, Table 1) were recorded daily through the first week of ICU stay. We extracted all daily TIL item values for our population, and calculated TIL_24_, uwTIL_24_, TIL^(Basic)^_24_, PILOT_24_, and TIL^(1987)^_24_ as defined in Table 1. For patients who underwent WLST after the first day of ICU stay, we only extracted TIL item information from before the documented date of WLST decision.

### ICP_EH_ and related values

End-hour ICP (ICP_EH_), systolic blood pressure (SBP_EH_), and diastolic blood pressure (DBP_EH_) were recorded by clinicians every 2 h for the TIL-ICP_EH_ sub-population. Mean arterial pressure (MAP_EH_) was calculated as MAP_EH_ = (SBP_EH_ + 2DBP_EH_)/3, and cerebral perfusion pressure (CPP_EH_) was calculated as CPP_EH_ = MAP_EH_ – ICP_EH_. From ICP_EH_ and CPP_EH_, we calculated the following values:

- ICP_24_ or CPP_24_, the mean ICP or CPP value over a calendar day of ICU stay,
- ICP_max_ or CPP_min_, the maximum ICP_24_ or minimum CPP_24_ value over the first week of a patient's ICU stay,
- ICP_median_ or CPP_median_, the median ICP_24_ or CPP_24_ value over the first week of a patient's ICU stay.

### ICP_HR_ and related values

High-resolution signals were collected using either ICM+ software (Cambridge Enterprise Ltd, Cambridge, U.K.; http://icmplus.neurosurg.cam.ac.uk), Moberg CNS monitor (Moberg Research Inc, Ambler, PA, USA; https://www.moberg.com), or both. Blood pressure was obtained through arterial lines connected to pressure transducers. High-resolution ICP (ICP_HR_) was acquired from either an intraparenchymal strain gauge probe (Codman ICP MicroSensor, Codman and Shurtleff Inc., Raynham, MA, USA), a parenchymal fiber optic pressure sensor (Camino ICP Monitor, Integra Life Sciences, Plainsboro, NJ, USA; https://www.integralife.com/), or an external ventricular drain. Detailed data collection and pre-processing methods (i.e., artefact cleaning and down-sampling to ten-second averaged time series) applied to high resolution signals in our study have been described previously.^21^ Ten-second averaged ICP (ICP_HR_10sec_) and CPP (CPP_HR_10sec_) time-series were retrieved for this analysis, and, from ICP_HR_10sec_ and CPP_HR_10s_, we calculated ICP_24_/CPP_24_, ICP_max_/CPP_min_, and ICP_median_/CPP_median_ as described above.

### Physician impressions

Attending ICU physicians were asked to record their daily concerns with the patient's ICP and CPP, separately, on a scale from 1 (not concerned) to 10 (most concerned). Moreover, on each patient's ICU discharge summary, physicians were asked to record whether the patient experienced refractory intracranial hypertension during his or her ICU stay. Refractory intracranial hypertension was defined as recurrent, sustained (i.e., of at least 10 min) increases of ICP above 20 mm Hg despite medical ICP management. We extracted the daily ICP/CPP concern ratings and refractory intracranial hypertension impressions which coincided with the ICU stays of our population.

### Baseline characteristics, prognosis, and outcome

We extracted baseline demographic characteristics, Marshall CT classifications,^22^ and Glasgow Coma Scale (GCS)^23^ scores from ICU admission.^24^ We also extracted Glasgow Outcome Scale—Extended (GOSE) functional outcome scores at 6 months post-injury,^25^ with imputation of missing values as previously described.^26^ Finally, we extracted ordinal functional outcome prognosis scores, calculated from a tokenized embedding of all available clinical information in the first 24 h of ICU stay, as described previously.^27^

### Validation

We appraised the validity of TIL according to recommendations of best practice from clinimetric literature.^28^ Based on the identified domain of TIL, we evaluated the construct and criterion validities of TIL. Our qualitative and quantitative assessments of TIL were performed against those of alternative scoring configurations (Table 1) for comparison.

### Construct validity

Construct validity is the extent to which a clinical scale matches expectations of associations with parameters within or outside the identified domain. Construct validity is further broken down into convergent validity (i.e., associations with similar constructs), discriminant validity (i.e., associations with divergent constructs), and differentiation by known groups. In this work, statistical associations between study variables were measured with:

- Spearman's correlation coefficients (*ρ*) for static (i.e., measured once) variables,
- repeated measures correlation coefficients (*r_rm_*)^29^—interpreted as within-individual strength of association—for longitudinal (i.e., measured over time) variables,
- linear mixed effects regression (LMER) coefficients (*β_LMER_*) of daily scale scores (e.g., TIL_24_) when regressing ICP_24_ or CPP_24_ on daily scale scores and the day of ICU stay (Day_ICU_), accounting for inter-patient variability with random intercepts. Therefore, *β_LMER_* were interpreted as the expected difference in ICP_24_ or CPP_24_ per unit increase of daily scale score, independent of time since ICU admission or inter-patient variation.

For convergent validity, we expected therapeutic intensity to correlate at least mildly (i.e., |*ρ*|≥0.2, |*r_rm_*|≥0.2, |*β_LMER_*|>0) with markers of injury severity (i.e., baseline GCS and baseline outcome prognoses), functional outcome (i.e., six-month GOSE), clinical concerns of ICP status, and ICP itself. Accordingly, we calculated: 1) *ρ* values between TIL_max_ and GCS, ordinal prognosis scores, GOSE, and ICP_max_; 2) *ρ* values between TIL_median_ and GCS, ordinal prognosis scores, GOSE, and ICP_median_; 3) *r_rm_* values between TIL_24_ and physician concern of ICP and ICP_24_; and 4) *β_LMER_* of TIL_24_ when regressing ICP_24_ on Day_ICU_ and TIL_24_ (i.e., ICP_24_∼Day_ICU_+TIL_24_), accounting for inter-patient variability with random intercepts.

For discriminant validity, we expected therapeutic intensity to be more strongly correlated with ICP and physician concerns of ICP than with CPP and physician concerns of CPP, respectively. Even though CPP control through fluid loading or vasopressor therapy is a component modality of TIL (Table 1), we expected TIL to capture ICP management (i.e., the construct) more accurately than CPP management. We compared: 1) *ρ* values of TIL_max_ versus CPP_min_ to those of TIL_max_ vs. ICP_max_; 2) *ρ* values of TIL_median_ versus CPP_median_ to those of TIL_median_ vs. ICP_median_; 3) *r_rm_* values of TIL_24_ versus CPP_24_ to those of TIL_24_ vs. ICP_24_; and 4) the *β_LMER_* of TIL_24_ when regressing CPP_24_∼Day_ICU_+TIL_24_ to the *β_LMER_* of TIL_24_ when regressing ICP_24_∼Day_ICU_+TIL_24_.

For differentiation by known groups, we expected TIL_max_ and TIL_median_ to effectively discriminate patients who experienced refractory intracranial hypertension during ICU stay from those who did not. We calculated the area under the receiver operating characteristic curve (AUC), which, in our case, was interpreted as the probability of a randomly selected patient with refractory intracranial hypertension having a higher TIL_max_ or TIL_median_ score than one without it. We also compared the AUCs of TIL_max_ and TIL_median_ to ICP_max_ and ICP_median_ and determined the sensitivity and specificity of refractory intracranial hypertension detection at each threshold of TIL_max_ and TIL_median_.

### Criterion validity

Criterion (or concurrent) validity is the degree to which there is an association between a clinical scale and other scales measuring the same construct, particularly a gold standard assessment. Since there is no extant “gold standard” for assessing ICP management intensity, we tested the concurrent criterion validity of TIL by calculating its associations with its predecessors (i.e., PILOT and TIL^(1987)^), mindful of their limitations as described above. More specifically, we calculated: 1) *ρ* values between TIL_max_ and prior scale maximum scores (i.e., PILOT_max_ and TIL^(1987)^_max_); 2) *ρ* values between TIL_median_ and prior scale median scores (i.e., PILOT_median_ and TIL^(1987)^_median_); and 3) *r_rm_* between TIL_24_ and prior scale daily scores (i.e., PILOT_24_ and TIL^(1987)^_24_).

### Component item analysis

We evaluated inter-item (i.e., inter-treatment) and adjusted item-total associations of TIL_24_, uwTIL_24_, PILOT_24_, and TIL^(1987)^_24_ by calculating *r_rm_* values. Item-total correlations were adjusted by subtracting the tested item score from the total score prior to calculating the correlation. We measured Cronbach's alpha (*α*) to assess internal reliability amongst scale items at each day of ICU stay. Moreover, we calculated the median score contribution of each item per total TIL_24_ score. The association between each TIL_24_ item score and ICP_24_, CPP_24_, physician concern of ICP, and physician concern of CPP was calculated with *r_rm_* values. Finally, we trained LMER models regressing ICP_24_ and CPP_24_ on all TIL items (with categorical dummy encoding) and Day_ICU_ concurrently. The *β_LMER_* values from these models were interpreted as the average change in ICP_24_ or CPP_24_ associated with each treatment when accounting for all other ICP-guided treatments, time since ICU admission, and inter-patient variability with random intercepts.

### TIL^(Basic)^ information coverage

We examined the distributions of TIL^(Basic)^_24_ per TIL_24_ and TIL_24_ per TIL^(Basic)^_24_ to derive thresholds for categorizing TIL_24_ into TIL^(Basic)^_24_. We also calculated the information coverage (IC) of TIL^(Basic)^ by dividing the mutual information (MI) of TIL^(Basic)^ and TIL by the entropy of TIL. IC was calculated with TIL^(Basic)^_24_ and TIL_24_ for days one through seven of ICU stay, with TIL^(Basic)^_max_ and TIL_max_, and with TIL^(Basic)^_median_ and TIL_median_.

### Statistical analysis

Multiple imputation of missing values. Five of the static study variables had missing values for some of the patients in our study: GCS, GOSE, baseline prognosis scores, Marshall CT classifications, and refractory intracranial hypertension status. We assessed the patterns of missingness (Supplementary Fig. S1) and multiply imputed (*m* = 100) these variables with independent, stochastic predictive mean matching functions using the mice package^30^ (v3.9.0) in R (v4.2.3). We assumed these variables to be missing-at-random (MAR; as previously reported on CENTER-TBI data)^31^ and supported this assumption by training imputation models on all study measures as well as correlated auxiliary variables (e.g., raised ICP during ICU stay).

For daily longitudinal study variables, we considered a value to be missing if the patient was still in the ICU and WLST had not been decided on or before that day. We assessed the longitudinal patterns of missingness (Supplementary Fig. S2) and multiply imputed (*m* = 100) these variables with the multivariate, time-series algorithm from the *Amelia II* package^32^ (v1.7.6) in R over the first week of ICU stay. The algorithm exploits both between-variable and within-variable correlation structures over time to stochastically impute missing time series values in independently trained runs. We validated the MAR assumption by identifying characteristics significantly associated with longitudinal variable missingness (Supplementary Table S1) and included auxiliary information associated with value missingness (e.g., reasons for stopping ICP monitoring) in the imputation model.

Statistical inference. We calculated 95% confidence intervals (CI) for ρ, r_rm_, β_LMER_, AUC, sensitivity, specificity, α, and IC values using bootstrapping with 1000 resamples of unique patients. For each resample, one of the 100 missing value imputations was randomly chosen. Therefore, confidence intervals represented the uncertainty due to patient resampling and missing value imputation.

### Code availability

All statistical analyses were performed in Python (v3.8.2) or R, and all visualizations were created in R. All scripts used in this study are publicly available on GitHub: https://github.com/sbhattacharyay/CENTER-TBI_TIL.

## Results

### Study population

Of the 4509 patients available for analysis in the CENTER-TBI core study, 873 patients from 52 ICUs met the additional inclusion criteria of this work. Amongst them, 837 constituted the TIL-ICP_EH_ sub-population and 259 constituted the TIL-ICP_HR_ sub-population (Fig. 1). Summary characteristics of the overall population as well as those of the TIL-ICP_EH_ and TIL-ICP_HR_ sub-populations are detailed in Table 2. Apart from two of the prognosis scores pertaining to the probability of returning to pre-injury life roles (i.e., Pr(GOSE >5) and Pr(GOSE >6)), none of the tested characteristics were significantly different between patients in the TIL-ICP_HR_ sub-population and those outside of it (Table 2).

**Table 2.** Summary Characteristics of Study Validation Populations

Summary characteristic	TIL validation population			
	Overall (*n* = 873, 52 centers)	TIL-ICP_EH_ (*n* = 837, 51 centers)	TIL-ICP_HR_ (*n* = 259, 21 centers)	*p* value^c^
Age [years]	47 (29–62)	47 (29–62)	48 (30–62.5)	0.303
Sex: Female	222 (25%)	213 (25%)	55 (21%)	0.078
Baseline GCS (*n*^a^ = 822)				0.554
Mild [13–15]	122 (15%)	115 (15%)	38 (16%)	
Moderate [9–12]	139 (17%)	133 (17%)	36 (15%)	
Severe [3–8]	561 (68%)	539 (68%)	170 (70%)	
Marshall CT (*n*^a^ = 710)				0.278
No visible pathology (I)	17 (2%)	16 (2%)	6 (3%)	
Diffuse injury II	264 (37%)	248 (36%)	75 (35%)	
Diffuse injury III	93 (13%)	89 (13%)	22 (10%)	
Diffuse injury IV	16 (2%)	16 (2%)	3 (1%)	
Mass lesion (V & VI)	320 (45%)	312 (46%)	107 (50%)	
Six-month GOSE (*n*^a^ = 761)				0.329
(1) Death	199 (26%)	195 (26%)	54 (23%)	
(2 or 3) Vegetative or lower SD	182 (24%)	181 (25%)	63 (27%)	
(4) Upper SD	70 (9%)	66 (9%)	22 (9%)	
(5) Lower MD	122 (16%)	117 (16%)	44 (19%)	
(6) Upper MD	74 (10%)	71 (10%)	23 (10%)	
(7) Lower GR	56 (7%)	52 (7%)	14 (6%)	
(8) Upper GR	58 (8%)	55 (7%)	13 (6%)	
Baseline functional prognosis^b^ [%] (*n*^a^ = 749)				
Pr(GOSE >1)	84.7 (63.5–94.9)	84.1 (62.1–94.7)	83.8 (66.9–94.0)	0.664
Pr(GOSE >3)	53.9 (29.9–76.0)	53.1 (29.2–75.0)	52.4 (33.9–71.1)	0.287
Pr(GOSE >4)	39.6 (20.6–59.6)	38.9 (19.8–58.3)	38.1 (22.6–54.6)	0.154
Pr(GOSE >5)	21.1 (10.2–36.8)	20.7 (10.0–36.0)	19.3 (10.5–30.1)	0.037
Pr(GOSE >6)	12.4 (5.9–20.8)	12.0 (5.8–19.9)	10.9 (5.8–17.2)	0.009
Pr(GOSE >7)	4.8 (2.2–9.2)	4.7 (2.2–9.1)	5.3 (2.2–8.5)	0.415
TIL_max_	10 (6–14)	10 (6–14)	10 (6–14)	0.577
TIL_median_	5 (3–10)	5 (3–10)	5 (4–10)	0.826
TIL_24_ scores				
Day 1 (*n*^a^ = 852)	7 (4–11)	7 (4–11)	7 (5–10)	0.134
Day 2 (*n*^a^ = 839)	6 (4–10)	6 (4–10)	6 (4–10)	0.860
Day 3 (*n*^a^ = 819)	6 (3–9)	6 (3–9)	6 (4–9)	0.926
Day 4 (*n*^a^ = 787)	6 (3–10)	6 (3–10)	5 (4–10)	0.372
Day 5 (*n*^a^ = 761)	5 (3–10)	5 (3–10)	5 (3–10)	0.941
Day 6 (*n*^a^ = 733)	5 (2–9)	5 (2.5–9)	5 (3–10)	0.337
Day 7 (*n*^a^ = 709)	5 (2–9)	4 (2–9)	5 (2–9)	0.425

^a^ Limited sample size of non-missing values for characteristic.

^b^ Ordinal functional outcome prognostic scores were calculated through tokenized embedding of all clinical information in the first 24 h of ICU stay, as described previously.^27^

^c^ The *p* values, comparing patients in TIL-ICP_HR_ sub-population to those not in TIL-ICP_HR_ sub-population, are derived from with Welch's *t*-test for numeric variables and χ^2^ contingency table test for categorical variables.

Data are median (interquartile range) for numeric characteristics and *n* (% of column group) for categorical characteristics, unless otherwise indicated. Units or numerical definitions of characteristics are provided in square brackets.

Baseline GCS, Glasgow Coma Scale at ICU admission, from 3 to 15; GOSE, Glasgow Outcome Scale-Extended; GR, good recovery; ICP, intracranial pressure; ICP_EH_, end-hour ICP; ICP_HR_, high-resolution ICP; Marshall CT, Marshall computerized tomography classification; MD, moderate disability; Pr(GOSE>•), “probability of GOSE greater than • at 6 months post-injury” as previously calculated from the first 24 h of admission^27^; SD, severe disability; TIL,Therapy Intensity Level scale; TIL_24_,TIL score of calendar day in ICU; TIL_max_, maximum TIL_24_ over first week of ICU stay; TIL_median_, median TIL_24_ over first week of ICU stay.

**FIG. 2.**
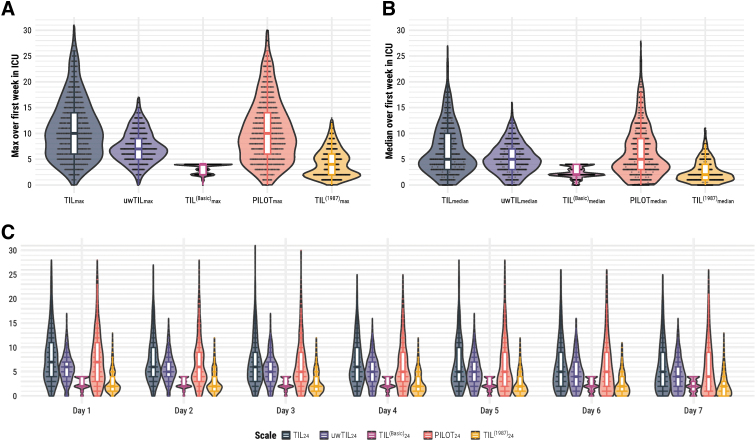
Distributions of TIL and alternative scales. The numeric definition of each scale is listed in Table 1. (**A**) Distributions of maximum scores of TIL (i.e., TIL_max_) and alternative scales (i.e., uwTIL_max_, TIL^(Basic)^_max_, PILOT_max_, and TIL^(1987)^_max_) over the first week of ICU stay. (**B**) Distribution of median scores of TIL (i.e., TIL_median_) and alternative scales (i.e., uwTIL_median_, TIL^(Basic)^_median_, PILOT_median_, and TIL^(1987)^_median_) over the first week of ICU stay. (**C**) Distributions of daily scores of TIL (i.e., TIL_24_) and alternative scales (i.e., uwTIL_24_, TIL^(Basic)^_24_, PILOT_24_, and TIL^(1987)^_24_) over the first week of ICU stay. ICU, intensive care unit; PILOT, Pediatric Intensity Level of Therapy scale^7^; TIL, Therapy Intensity Level scale^8,9^; TIL^(1987)^, original Therapy Intensity Level scale published in 1987^6^; TIL^(Basic)^, condensed TIL scale^8^; uwTIL, unweighted TIL scale in which sub-item scores are replaced by the ascending rank index within the item.

The median ICU stay duration of our population was 14 days (IQR: 7.8–23 days), and 83% (*n* = 726) stayed through at least seven calendar days. At each day of ICU stay, less than 2.4% of the expected TIL scores were missing (Supplementary Fig. S2). Each TIL component item (Table 1) is represented by at least 17% (*n* = 147, intracranial surgery) and each sub-item is represented by at least 4.9% (*n* = 43, high-dose mannitol) of the population (Supplementary Table S2). The distributions of TIL_max_, TIL_median_, and TIL_24_, juxtaposed against the scores of alternative scales (Table 1), are displayed in Figure 2. The distributions of TIL and PILOT were visually similar, and TIL^(Basic)^_max_ had a strong ceiling effect (i.e., 57% of the population had the maximum score). Whilst there was no significant difference in TIL_24_ distribution over the first seven days, most patients had their highest TIL_24_ (i.e., TIL_max_) soon after ICU admission (median: day two, IQR: days one–three). The Spearman's rank correlation coefficient (*ρ*) between TIL_max_ and TIL_median_ was 0.80 (95% CI: 0.77–0.82), and the median TIL_median_:TIL_max_ ratio was 0.65 (IQR: 0.45–0.80).

### Validation of TIL

**FIG. 3.**
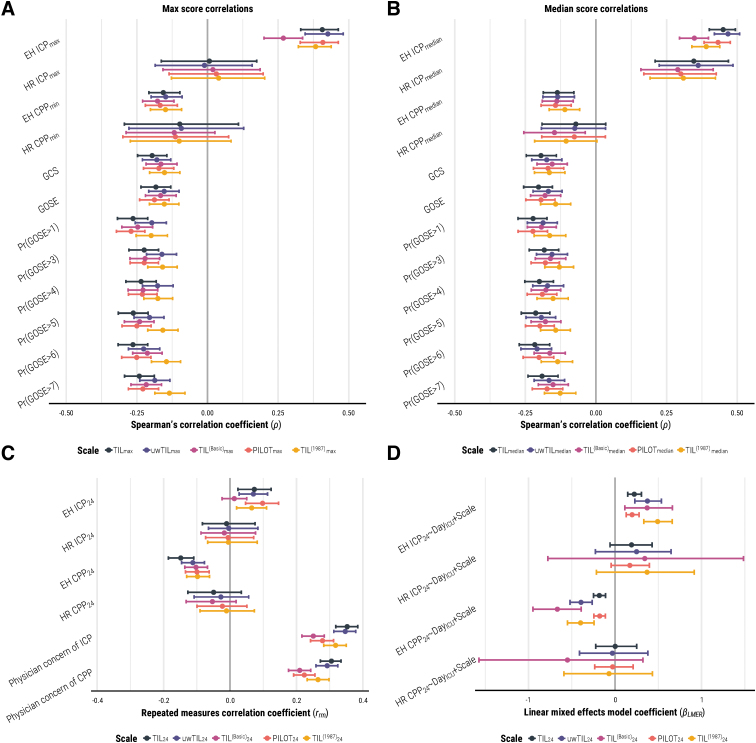
Associations of TIL and alternative scales with other clinical measures. The numeric definition of each scale is listed in Table 1, and the calculation of daily (e.g., TIL_24_), maximum (e.g., TIL_max_), and median (e.g., TIL_median_) scores are described in the “Methods” Section. The bars represent 95% confidence intervals derived from bootstrapping with 1,000 resamples of unique patients over 100 missing value imputations. (**A**) Spearman's correlation coefficients (*ρ*) between maximum scale scores over first week of ICU stay (i.e., TIL_max_, uwTIL_max_, TIL^(Basic)^_max_, PILOT_max_, and TIL^(1987)^_max_) and other clinical measures. (**B**) Spearman's correlation coefficients (*ρ*) between median scale scores over first week of ICU stay (i.e., TIL_median_, uwTIL_median_, TIL^(Basic)^_median_, PILOT_median_, and TIL^(1987)^_median_) and other clinical measures. (**C**) Repeated measures correlation coefficients (*r_rm_*, from -1 to 1) are interpreted as the strength and direction of association between two variables after accounting for inter-patient variation. (**D**) Linear mixed effects model coefficients (*β_LMER_*) are interpreted as the expected difference in dependent variable (e.g., EH ICP_24_) per unit increase of daily scale score (e.g., TIL_24_) after accounting for time since ICU admission (i.e., Day_ICU_) and inter-patient variation. Day_ICU_, variable representing day (from 1 to 7) of ICU stay; EH, end-hour; CPP, cerebral perfusion pressure; GCS, Glasgow Coma Scale at ICU admission; GOSE, Glasgow Outcome Scale–Extended at 6 months post-injury; HR, high-resolution; ICP, intracranial pressure; ICU, intensive care unit; PILOT, Pediatric Intensity Level of Therapy scale^7^; Pr(GOSE>•), “probability of GOSE greater than • at 6 months post-injury” as previously calculated from the first 24 h of admission^27^; TIL, Therapy Intensity Level scale^8,9^; TIL^(1987)^, original Therapy Intensity Level scale published in 1987^6^; TIL^(Basic)^, condensed TIL scale^8^; uwTIL, unweighted TIL scale in which sub-item scores are replaced by the ascending rank index within the item.

**FIG. 4.**
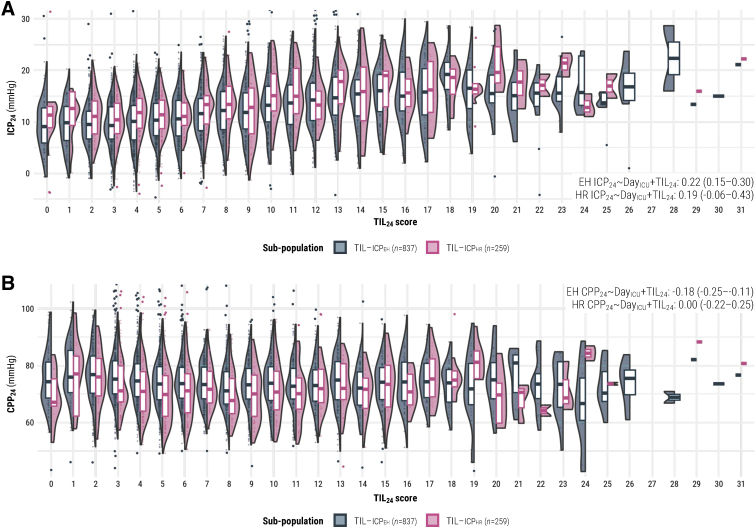
Distributions of daily intracranial pressure and cerebral perfusion pressure means per daily TIL score. The values in each panel are the linear mixed effects model coefficients (*β_LMER_*) of TIL_24_ with 95% confidence intervals derived from bootstrapping with 1000 resamples of unique patients over 100 missing value imputations. The width of violin plots is scaled for each population, but the width of the points inside them demonstrates relative frequency across the populations. The violin plots do not encompass outliers based on 1.5 times the interquartile range. (**A**) Distributions of ICP_24_ vs. TIL_24_ for both sub-populations. (**B**) Distributions of CPP_24_ vs. TIL_24_ for both sub-populations. CPP, cerebral perfusion pressure; CPP_24_, mean CPP over calendar day; Day_ICU_, variable representing day (from 1 to 7) of ICU stay; EH, end-hour; HR, high-resolution; ICP, intracranial pressure; ICP_24_, mean ICP over calendar day; TIL, Therapy Intensity Level scale^8,9^; TIL_24_, TIL score of calendar day; TIL-ICP_EH_, end-hour ICP sub-population; TIL-ICP_HR_, high-resolution ICP sub-population.

The 95% CIs of *ρ* values, repeated measures correlation coefficients (*r_rm_*), and linear mixed effect regression coefficients (*β_LMER_*) of TIL with other study measures are visualized in Fig. 3. Both TIL_max_ and TIL_median_ had mildly negative correlations (-0.26 < *ρ*_mean_ < -0.19) with baseline GCS, six-month GOSE, and functional outcome prognoses (Fig. 3A, 3B). The within-individual association of TIL_24_ with physician concerns of ICP was moderately positive (*r_rm_ =* 0.35 [95% confidence interval [CI]: 0.31-0.38]) and significantly higher than that of TIL^(Basic)^_24_ (Fig. 3C). The association between ICP_median_ and TIL_median_ was moderately positive (0.35 < *ρ*_mean_ < 0.45) with both ICP_EH_ and ICP_HR_ values, and the association between ICP_max_ and TIL_max_ was moderately positive (*ρ =* 0.41 [95% CI: 0.33-0.46]) with ICP_EH_ values. The ICP_max_ vs. TIL_max_ correlation was not significant (*ρ =* 0.01 [95% CI: -0.16-0.17]) with ICP_HR_ values; however, without imputing missing ICP_HR_ values, the *ρ* was 0.43 (95% CI: 0.35-0.50). This suggests that the longitudinal missingness of ICP_HR_ (Supplementary Fig. S2) for our sample size made the ICP_max_ estimation significantly imprecise. Additionally, the within-individual association with ICP_24_ was either weak or not significant for any daily scale score according to *r_rm_* (Fig. 3C) and *β_LMER_* (Fig. 3D) values. On average, a single point increase in TIL_24_ was associated with a 0.22 (95% CI: 0.15–0.30) mm Hg increase in daily mean ICP_EH_ and a 0.19 (95% CI: -0.06-0.43) mm Hg increase in daily mean ICP_HR_. These results mostly affirm the convergent validity of TIL but highlight the broad intra-patient variability between ICP and therapeutic intensity. From the distribution of ICP_24_ values at each TIL_24_ score (Fig. 4A), we observed both considerable ICP_24_ overlap across each TIL_24_ score and an overall positive relationship between TIL_24_ and ICP_24_, particularly for TIL_24_ ≥ 8.

The correlation between TIL and both prior scales (i.e., PILOT and TIL^(1987)^) was positively strong for maximum, median, and daily scores (Supplementary Fig. S3), establishing the criterion validity of TIL. According to 95% CIs, the association of TIL with prior scales was stronger than that of uwTIL or TIL^(Basic)^ (Supplementary Fig. S3).

According to *ρ*, *r_rm_*, and *β_LMER_* values (Fig. 3), the associations of TIL with CPP and of TIL with physician concerns of CPP were weaker than or not significantly different from the corresponding associations with ICP. Moreover, the trend of CPP_24_ distributions over different TIL_24_ scores is not as visually apparent as that of ICP_24_ distributions over different TIL_24_ scores (Fig. 4B). These results support the discriminant validity of TIL.

**FIG. 5.**
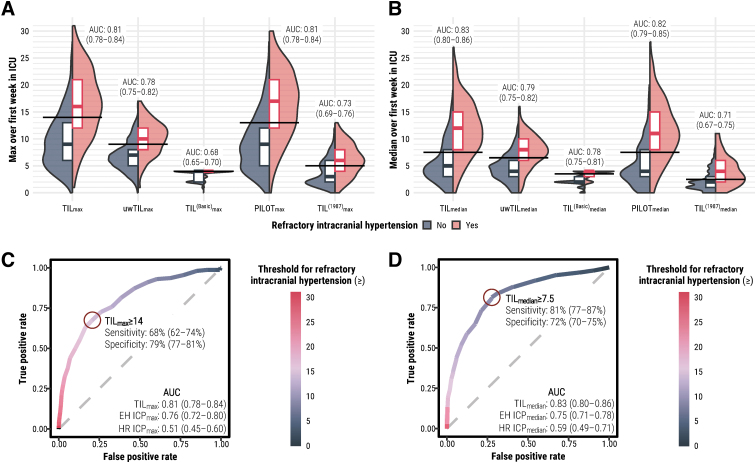
Discrimination of refractory intracranial hypertension status by TIL and alternative scale summary scores. The 95% confidence intervals of AUC were derived from bootstrapping with 1,000 resamples of unique patients over 100 missing value imputations. (**A**) Distributions of maximum scores of TIL (i.e., TIL_max_) and alternative scales (i.e., uwTIL_max_, TIL^(Basic)^_max_, PILOT_max_, and TIL^(1987)^_max_) stratified by refractory intracranial hypertension status. The horizontal black line segments represent the thresholds which maximized the sum of sensitivity and specificity for each scale. (**B**) Distributions of median scores of TIL (i.e., TIL_median_) and alternative scales (i.e., uwTIL_median_, TIL^(Basic)^_median_, PILOT_median_, and TIL^(1987)^_median_) stratified by refractory intracranial hypertension status. The horizontal black line segments represent the thresholds which maximized the sum of sensitivity and specificity for each scale. (**C**) Receiver operating characteristic curve of refractory intracranial hypertension detection with TIL_max_. The threshold which maximized the sum of sensitivity and specificity is highlighted with the dark red circle. (**D**) Receiver operating characteristic curve of refractory intracranial hypertension detection with TIL_median_. The threshold which maximized the sum of sensitivity and specificity is highlighted with the dark red circle. AUC, area under the receiver operating characteristic curve, EH, end-hour; HR, high-resolution; ICP, intracranial pressure; ICP_max_, maximum calendar day mean of ICP over first week of ICU stay; ICP_median_, median calendar day mean of ICP over first week of ICU stay; ICU, intensive care unit; PILOT, Pediatric Intensity Level of Therapy scale^7^; TIL, Therapy Intensity Level scale^8,9^; TIL^(1987)^, original Therapy Intensity Level scale published in 1987^6^; TIL^(Basic)^, condensed TIL scale^8^; uwTIL, unweighted TIL scale in which sub-item scores are replaced by the ascending rank index within the item.

In our population, 157 patients (18% of 864 assessed) were reported to experience refractory intracranial hypertension during ICU stay. TIL_max_ correctly discriminated these patients from the others 81% (95% CI: 78-84%) of the time (Fig. 5A), and TIL_median_ did so 83% (95% CI: 80-86%) of the time (Fig. 5B). This performance of TIL was significantly greater than or similar to that of all alternative scales (Fig. 5A, 5B). Further, TIL_median_ had significantly greater discrimination performance than ICP_max_ (Fig. 5C) and ICP_median_ (Fig. 5D), respectively. The sensitivity and specificity of refractory intracranial hypertension detection at each threshold of TIL_max_, TIL_median_, TIL^(Basic)^_max_, and TIL^(Basic)^_median_ are listed in Supplementary Table S3 and visualized in Figure 5C and 5D. The thresholds which maximized the sum of sensitivity and specificity were TIL_max_ ≥ 14 (sensitivity: 68% [95% CI: 62–74%], specificity: 79% [95% CI: 77-81%]) and TIL_median_ ≥ 7.5 (sensitivity: 81% [95% CI: 77-87%], specificity: 72% [95% CI: 70-75%]; Table 3).

**Table 3.** Optimized Ranges for TIL Categorization

Category	Derived ranges	Performance (95% confidence intervals)			Case counts^c^		Previously proposed ranges^d^
		Sensitivity	Specificity	Accuracy	No	Yes	
Refractory intracranial hypertension^a^	TIL_max_≥14	68% (62–74%)	79% (77–81%)	77% (75–79%)	707	157	TIL_max_≥11
	TIL_median_≥7.5	81% (77–87%)	72% (70–75%)	74% (72–76%)			–
Day of surgical ICP control^b^	TIL_24_≥9	87% (83–91%)	74% (72–76%)	76% (74–77%)	4916	585	–
TIL^(Basic)^_24_				72% (70–73%)			
(1) Basic ICU care	1≤TIL_24_≤2				4932	568	1≤TIL_24_≤3
(2) Mild	3≤TIL_24_≤6				3294	2206	4≤TIL_24_≤7
(3) Moderate	7≤TIL_24_≤8				4709	791	8≤TIL_24_≤10
(4) Extreme	TIL_24_≥9				3919	1581	TIL_24_≥11

The numeric definition of each scale is listed in Table 1, and the calculation of daily (e.g., TIL_24_), maximum (e.g., TIL_max_), and median (e.g., TIL_median_) scores is described in the Methods. The 95% confidence intervals of performance metrics were derived from bootstrapping with 1000 resamples of unique patients over 100 missing value imputations.

^a^ Refractory intracranial hypertension was defined as recurrent, sustained (i.e., of at least 10 min) increases of ICP above 20 mm Hg despite medical ICP management during ICU stay. This information was recorded by attending physicians in patient discharge summaries.

^b^ If a decompressive craniectomy was performed as a last resort for refractory intracranial hypertension, each of the days following the operation were also considered days of surgical ICP control.

^c^ For refractory intracranial hypertension, case counts represent the number of patients (with non-missing values) without (i.e., No) and with (i.e., Yes) refractory intracranial hypertension. For day of surgical ICP control and TIL^(Basic)^_24_, case counts represent the number of non-missing TIL assessments not in (i.e., No) and in (i.e., Yes) the given category.

^d^ Thresholds were previously proposed by the interagency panel which developed TIL based on expert opinion.^8^

ICP, intracranial pressure; ICU, intensive care unit; TIL, Therapy Intensity Level scale^8,9^; TIL^(Basic)^, condensed TIL scale.^8^

### TIL component items

**FIG. 6.**
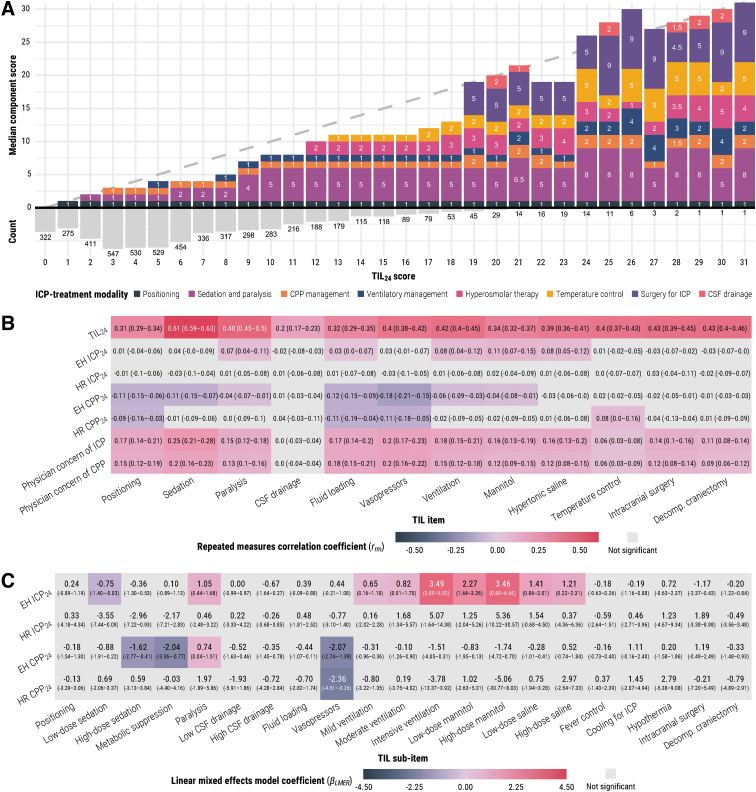
Association of TIL component items with TIL_24_ and other study measures. The 95% confidence intervals of *r_rm_* and *β_LMER_* values were derived from bootstrapping with 1000 resamples of unique patients over 100 missing value imputations. (**A**) Median component score of each ICP-treatment modality (Table 1) per each TIL_24_ score. The histogram under the *x*-axis represents the relative frequency and count of each TIL_24_ score in the population, and diagonal dashed line represents the TIL_24_ score on both axes. If the sum of median item scores does not equal the corresponding TIL_24_ score, this can be interpreted as high variability in the combination of simultaneously applied therapies at that TIL_24_ score. (**B**) The repeated measures correlation coefficients (*r_rm_*, from -1 to 1) are interpreted as the strength and direction of association between two variables after accounting for inter-patient variation. The component score of each item (Table 1, *x*-axis) was subtracted from the TIL_24_ score (top row on *y*-axis) before calculating their *r_rm_* values. (**C**) Linear mixed effects model coefficients (*β_LMER_*) are interpreted as the expected difference in the dependent variable (*y*-axis) associated with the given TIL_24_ sub-item treatment (Table 1) after accounting for all other TIL_24_ sub-items, time since ICU admission, and inter-patient variation. CPP, cerebral perfusion pressure; CPP_24_, mean CPP over calendar day; CSF, cerebrospinal fluid; EH, end-hour; HR, high-resolution; ICP, intracranial pressure; ICP_24_, mean ICP over calendar day; ICU, intensive care unit; TIL, Therapy Intensity Level scale^8,9^; TIL_24_, TIL score of calendar day.

While there was wide variation in item combinations per TIL_24_ score (i.e., sum of median scores was often under diagonal line in Fig. 6A), the average order of therapeutic escalation was fairly consistent: position, sedation, CPP management, ventilatory management, neuromuscular blockade, hyperosmolar therapy, temperature control, and then surgery for refractory ICP. Surgical control of ICP occurred in over 50% of reported cases at each TIL_24_ above 18 (Fig. 6A), but the threshold which maximized the sum of sensitivity and specificity in detecting surgical ICP control was TIL_24_ ≥ 9 (Table 3, performance at each threshold is listed in Supplementary Table S4).

The inter-item *r_rm_* values of TIL_24_ (Supplementary Fig. S4) were mostly positive except for cerebrospinal fluid (CSF) drainage, which did not correlate significantly with most other items, and decompressive craniectomy, which did not correlate significantly with CSF, ventilatory, or temperature control. Consistent with Fig. 6A, this result suggested that CSF drainage and decompressive craniectomy were the most variably applied therapies across study ICUs. The Cronbach's alpha (*α*) value of TIL_24_ was, at best, 0.65 (95% CI: 0.62-0.68) and lower (albeit, not significantly) than that of uwTIL_24_ at each day of ICU stay (Supplementary Fig. S5). However, since TIL is a formative scale (i.e., the construct is multi-dimensional and defined by the items), high inter-item correlation and *α* values are not necessary for item validation.^17^ Among all TIL_24_ items, sedation was most strongly correlated with adjusted TIL_24_ scores and physician concerns of ICP (Fig. 6B). From 10 ≤ TIL_24_ ≤ 20, a plateau effect of high-dose sedation combined with neuromuscular blockade was observed in most cases (Fig. 6A). When accounting for all other TIL_24_ sub-items, time since ICU admission, as well as inter-patient variability, ventilation, mannitol administration, and hypertonic saline administration were most strongly associated with ICP_24_ and vasopressors were most strongly associated with CPP_24_ (Fig. 6C).

### TIL^(Basic)^

**FIG. 7.**
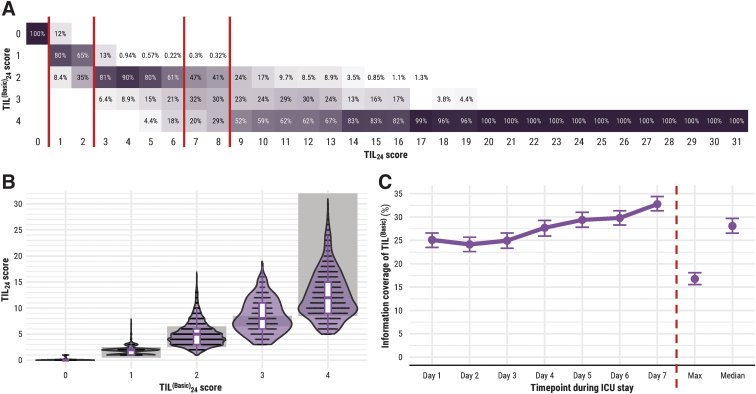
Relationship between TIL and TIL^(Basic)^. The numeric definition of each scale is listed in Table 1, and the calculation of daily (e.g., TIL_24_), maximum (e.g., TIL_max_), and median (e.g., TIL_median_) scores are described in the “Methods” section. The 95% confidence intervals of information coverage were derived from bootstrapping with 1000 resamples of unique patients over 100 missing value imputations. (**A**) Distribution of corresponding TIL^(Basic)^_24_ scores per each TIL_24_ score. The values in each cell represent the percent of assessments at a given TIL_24_ score (i.e., column) corresponding to a TIL^(Basic)^_24_ score (i.e., row). The vertical, dark red lines represent cut-offs across which the median corresponding TIL^(Basic)^_24_ score per TIL_24_ score changes. (**B**) Distribution of corresponding TIL_24_ scores per each TIL^(Basic)^_24_ score. The width of violin plots is scaled for each TIL^(Basic)^_24_ score, but the width of the points inside them demonstrates relative frequency across the TIL^(Basic)^_24_ scores. The grey, shaded zones represent the range of TIL_24_ scores with corresponding median TIL^(Basic)^_24_ scores on the *x*-axis, as determined in panel (A). (**C**) The information of TIL_24_, TIL_max_, and TIL_median_ covered by TIL^(Basic)^_24_, TIL^(Basic)^_max_, and TIL^(Basic)^_median_, respectively. Information coverage is defined as the mutual information of TIL_24_ and TIL^(Basic)^_24_ (or TIL_max_ and TIL^(Basic)^_max_ or TIL_median_ and TIL^(Basic)^_median_) divided by the entropy of TIL_24_ (or TIL_max_ or TIL_median_). AUC, area under the receiver operating characteristic curve; ICU, intensive care unit; TIL, Therapy Intensity Level scale^8,9^; TIL^(Basic)^, condensed TIL scale.^8^

Based on the median TIL^(Basic)^_24_ score at each TIL_24_ score (Fig. 7A), we derived the ranges for mapping TIL_24_ onto TIL^(Basic)^_24_ in Table 3. There is, however, considerable overlap of TIL_24_ scores across TIL^(Basic)^_24_ scores (Fig. 7B), particularly in the range of 6 ≤ TIL_24_ ≤ 10. TIL^(Basic)^_24_ = 3 was not the most represented score at any TIL_24_ score (Fig. 7A). TIL^(Basic)^_24_ covered up to 33% (95% CI: 31-34%) of the information (i.e., entropy) in TIL_24_, and TIL^(Basic)^_median_ covered up to 28% (95% CI: 27-30%) of the information in TIL_median_ (Fig. 7C). TIL^(Basic)^_max_ only covered 17% (95% CI: 16-18%) of the information in TIL_max_ (Fig. 7C).

## Discussion

In this work, we performed a large-scale (*n* = 873), multi-center (52 ICUs, 19 countries), and prospective validation study of TIL and TIL^(Basic)^ against alternative scales. Our results support the validity of TIL as a metric for scoring ICP-directed therapeutic intensity. The dataset we used, as part of the CENTER-TBI study, not only reflects the modern variation in ICP-directed therapeutic intensity (Fig. 2 and Fig. 6A) but also illustrates the practical feasibility of daily TIL assessment: of 885 eligible patients, 873 (99%) had daily TIL scores (Fig. 1) with less than 2.4% daily missingness (Supplementary Fig. S2).

We scrutinized and validated the use of TIL as a metric for scoring ICP-directed therapeutic intensity and for marking pathophysiological severity. The statistical construct and criterion validity measures of TIL were significantly greater or similar to those of alternative scales (Fig. 3 and Fig. 5), and TIL integrated the widest range of modern ICP treatments (Table 1). Summarized TIL scores outperformed summarized ICP values in detecting refractory intracranial hypertension. Our analysis yielded empirical ranges for interpreting TIL in terms of refractory intracranial hypertension, surgical intervention, and the condensed, TIL^(Basic)^ scores (Table 3). On a component level (Fig. 6A), TIL_24_ reflected a pattern of treatment intensity escalation consistent with clinical algorithms^2,3,5^ as well as a wide variation in treatment combinations, particularly in the use of CSF drainage and decompressive craniectomy. This finding is consistent with a previous CENTER-TBI study—which revealed inter-center variation in TIL treatment selection and time to administration^12^—and encourages an investigation of differences in TIL and long-term outcome between centers with known differences in ICP management strategies. In summary, our results support the use of TIL as an intermediate outcome for treatment effect, as done in previous studies.^33-35^

Due to a strong ceiling effect (Fig. 2A and Fig. 5A), TIL^(Basic)^ should not be used instead of TIL for rating maximum treatment intensity. TIL^(Basic)^_24_ and TIL_median_ covered up to 33% of the information in TIL_24_ (Fig. 7C), but the TIL^(Basic)^_24_ associations with physician concerns of ICP were significantly worse than those of TIL_24_ (Fig. 3C). TIL should always be preferred to TIL^(Basic)^, but we believe daily or median TIL^(Basic)^ can be a suitable alternative when daily or median TIL assessment is infeasible.

Moreover, we evaluated TIL with both end-hour (ICP_EH_) and high-resolution (ICP_HR_) ICP values. ICP_HR_, if available, should be considered the gold standard in terms of precision and granularity of the information provided, and neuromonitoring-related results from the ICP_HR_ population should generally take precedence.^14^ However, 67% of expected ICP_HR_ values were missing on Day 1 of ICU stay (Supplementary Fig. S2), likely due to the time required to arrange high-resolution data collection. Consequently, estimates of high-resolution ICP_max_ were significantly affected by missing value imputation and became imprecise at our sample size (Fig. 3A). In these cases, results from the ICP_EH_ population served as a valuable reference on a substantially larger sample size (Table 2) since ICP_EH_ and CPP_EH_ have been shown to be fair end-hour representations of ICP_HR_ and CPP_HR_, respectively, in CENTER-TBI.^14^ The considerable overlap of ICP_24_ values across TIL_24_ scores (both at low and high levels of ICP, Fig. 4A) and the insignificant-to-weak within-individual association between ICP_24_ and TIL_24_ (Fig. 3C–D) highlight the need to account for therapeutic intensity when interpreting ICP. Additionally, the higher median ICP_24_ values for TIL_24_ ≥ 8 (Fig. 4A) may suggest that clinicians accept a slightly higher ICP when balancing the risks of elevating therapeutic intensity against those of intracranial hypertension.

We see three main opportunities to improve TIL. First, the item scores of TIL and its predecessors (i.e., PILOT and TIL^(1987)^) were not derived empirically. Data-driven techniques, such as confirmatory factor analysis,^28^ can be used to derive scoring configurations, which optimize a defined objective (e.g., maximal separation of patients). However, data-driven scores do not necessarily reflect the intended construct (i.e., treatment risk and complexity),^36^ and, in general, item scoring does not have an appreciable impact on overall scale performance.^28^ Second, the items of TIL must evolve as therapeutic approaches to ICP management evolve. TIL discriminated refractory intracranial hypertension status significantly better than TIL^(1987)^ (Fig. 5A, 5B) because TIL updated TIL^(1987)^ with six additional items (Table 1). We recommend updating and re-evaluating TIL each time ICP-treatment modalities or their perceived risks change. Finally, the development of TIL was largely informed by the perspective of ICU practices in high-income countries.^8^ Likewise, this assessment was performed in a cohort of patients across Europe and Israel. Especially given the disproportionately higher burden of TBI in low- and middle-income countries,^37^ it is imperative to test and, if necessary, adapt TIL to a more inclusive, global population of TBI.

By design, TIL does not encompass all facets of modern intensive care for TBI patients. Brain tissue oxygen tension (PbtO_2_),^38^ cerebral microdialysis,^39^ and brain temperature^40^ have emerged as multi-modal neuromonitoring targets that may affect ICU management in addition to ICP or CPP. Therefore, TIL should be interpreted not as general treatment intensity but rather as the intensity of ICP-directed therapy specifically. We encourage the development and validation of clinical scales assessing the intensity of TBI treatments directed at other physiological targets. Since treatments for other targets often overlap with those for ICP or CPP (e.g., vasopressors target both PbtO_2_ and CPP),^2^ we also promote a consolidation of all TBI treatments in an overall therapeutic intensity scale which considers the effect of each treatment on multiple physiological targets.

We recognize several limitations of our analysis. Whilst numerous investigators assessed TIL across the study ICUs, each TIL score was only assessed once. Therefore, we could not evaluate the inter-rater reliability of TIL. Similarly, data needed to calculate the full TIL score were only recorded once a day, so we could not determine if a daily assessment frequency was sufficient. Since the prior TIL validation study reported a high inter-rater reliability and recommended a daily assessment frequency,^9^ we assumed both to be true. The results from the Randomized Evaluation of Surgery with Craniectomy for Uncontrollable Elevation of Intracranial Pressure (RESCUEicp) trial^41^—published amidst CENTER-TBI patient recruitment in 2016—have likely changed the global frequency and perceived intensity of decompressive craniectomy for TBI. Therefore, we recognize the potentially confounding effect of the trial results on treatment decision making for some patients in the CENTER-TBI population and encourage a potential reappraisal of the therapeutic intensity of decompressive craniectomy through expert discussion and statistical validation. The physician impressions (i.e., physician concerns of ICP and CPP and refractory intracranial hypertension status) were subjective, and we did not have enough information to account for inter-rater variability. Therefore, these scores and labels should be considered unrefined. Finally, because of limited dosage data for numerical treatments (i.e., CSF drainage, ventilation, hyperosmolar therapy, and temperature control), we did not test alternative sub-item categorizations.

## Conclusion

TIL is a valid, generalizable measurement of ICP management amongst neuro-monitored TBI patients in the ICU. On all validation metrics, TIL performs at least as well as its alternatives and considers the widest range of modern treatment strategies. TIL's component scores over increasing TIL reflect a clinically credible order of treatment escalation, from head positioning to ICP-directed surgery. TIL^(Basic)^ is not suitable for evaluating maximum treatment intensity, but daily TIL^(Basic)^ and median TIL^(Basic)^ can cover up to a third of the information in TIL. In the setting of clinical ICP management, TIL is a more sensitive marker of pathophysiological severity than ICP and can be considered an intermediate outcome after TBI.

## Transparency, Rigor, and Reproducibility Summary

The CENTER-TBI study was pre-registered at clinicaltrials.gov (NCT02210221, https://clinicaltrials.gov/ct2/show/NCT02210221). The analysis plan was registered after beginning data collection but before data analysis at https://www.center-tbi.eu/data/approved-proposals (#491), and the lead author with primary responsibility for the analysis certifies that the analysis plan was pre-specified. A sample size of 903 patients was planned based on availability of critically ill, ICP-monitored, adult TBI patients recruited for CENTER-TBI. Actual sample size was 873, as 18 patients had a documented decision to WLST on the first day of ICU stay and 12 additional patients did not have daily TIL scores assessed. A patient inclusion diagram is provided (Fig. 1). TIL scoring and clinical data entry was performed by investigators who were aware of relevant characteristics of the participants. Participants were recruited between December 19, 2014, and December 17, 2017, and data (including follow-up results) were collected until March 31, 2021. High-resolution waveforms were stored directly from bedside monitoring software, as described in the “Methods” section. Variability amongst different TIL assessors is not expected to be significant based on the established high inter-rater reliability of TIL.^9^ All equipment and software used to perform imaging and preprocessing are widely available from commercial sources or open source repositories. The clinimetric validation procedure and the primary clinical metric (TIL) are established standards in the field, based on previously published results^9,28^ and this study. The assumption of bootstrapping-derived confidence intervals is that the sample is representative of the population. This study is, itself, an external validation, and internal replication by the study group was performed. Individual participant data are available online, conditional to approved online study proposal, with no end date at https://www.center-tbi.eu/data. Signed confirmation of a data access agreement is required, and all access must comply with regulatory restrictions imposed on the original study. All analytic code used to perform the statistical analyses are publicly available online at https://github.com/sbhattacharyay/CENTER-TBI_TIL. This paper will be published under a Creative Commons Open Access license, and upon publication, will be freely available at https://www.liebertpub.com/loi/neu.

## Supplementary Material

Supplemental data

Supplemental data

Supplemental data

Supplemental data

Supplemental data

Supplemental data

Supplemental data

Supplemental data

Supplemental data

## The CENTER-TBI Investigators and Participants

Cecilia Åkerlund, Department of Physiology and Pharmacology, Section of Perioperative Medicine and Intensive Care, Karolinska Institutet, Stockholm, Sweden; Krisztina Amrein, János Szentágothai Research Center, University of Pécs, Pécs, Hungary; Nada Andelic, Division of Clinical Neuroscience, Department of Physical Medicine and Rehabilitation, Oslo University Hospital and University of Oslo, Oslo, Norway; Lasse Andreassen, Department of Neurosurgery, University Hospital Northern Norway, Tromso, Norway; Audny Anke, Department of Physical Medicine and Rehabilitation, University Hospital Northern Norway, Tromso, Norway; Anna Antoni, Trauma Surgery, Medical University Vienna, Vienna, Austria; Gérard Audibert, Department of Anesthesiology and Intensive Care, University Hospital Nancy, Nancy, France; Philippe Azouvi, Raymond Poincare hospital, Assistance Publique—Hopitaux de Paris, Paris, France; Maria Luisa Azzolini, Department of Anesthesiology and Intensive Care, S Raffaele University Hospital, Milan, Italy; Ronald Bartels, Department of Neurosurgery, Radboud University Medical Center, Nijmegen, the Netherlands; Pál Barzó, Department of Neurosurgery, University of Szeged, Szeged, Hungary; Romuald Beauvais, International Projects Management, ARTTIC, Munich, Germany; Ronny Beer, Department of Neurology, Neurological Intensive Care Unit, Medical University of Innsbruck, Innsbruck, Austria; Bo-Michael Bellander, Department of Neurosurgery and Anesthesia and Intensive Care Medicine, Karolinska University Hospital, Stockholm, Sweden; Antonio Belli, NIHR Surgical Reconstruction and Microbiology Research Center, Birmingham, U.K.; Habib Benali, Anesthesie-Réanimation, Assistance Publique—Hopitaux de Paris, Paris, France; Maurizio Berardino, Department of Anesthesia and ICU, AOU Città della Salute e della Scienza di Torino—Orthopedic and Trauma Center, Torino, Italy; Luigi Beretta, Department of Anesthesiology and Intensive Care, S Raffaele University Hospital, Milan, Italy; Morten Blaabjerg, Department of Neurology, Odense University Hospital, Odense, Denmark; Peter Bragge, BehaviourWorks Australia, Monash Sustainability Institute, Monash University, Victoria, Australia; Alexandra Brazinova, Department of Public Health, Faculty of Health Sciences and Social Work, Trnava University, Trnava, Slovakia; Vibeke Brinck, Quesgen Systems Inc., Burlingame, California, USA; Joanne Brooker, Australian and New Zealand Intensive Care Research Center, Department of Epidemiology and Preventive Medicine, School of Public Health and Preventive Medicine, Monash University, Melbourne, Australia; Camilla Brorsson, Department of Surgery and Perioperative Science, Umeå University, Umeå, Sweden; Andras Buki, Department of Neurosurgery, Medical School, University of Pécs, Hungary and Neurotrauma Research Group, János Szentágothai Research Center, University of Pécs, Hungary; Monika Bullinger, Department of Medical Psychology, Universitätsklinikum Hamburg-Eppendorf, Hamburg, Germany; Manuel Cabeleira, Brain Physics Lab, Division of Neurosurgery, Department of Clinical Neurosciences, University of Cambridge, Addenbrooke's Hospital, Cambridge, U.K.; Alessio Caccioppola, Neuro ICU, Fondazione IRCCS Cà Granda Ospedale Maggiore Policlinico, Milan, Italy; Emiliana Calappi, Neuro ICU, Fondazione IRCCS Cà Granda Ospedale Maggiore Policlinico, Milan, Italy; Maria Rosa Calvi, Department of Anesthesiology and Intensive Care, S Raffaele University Hospital, Milan, Italy; Peter Cameron, ANZIC Research Center, Monash University, Department of Epidemiology and Preventive Medicine, Melbourne, Victoria, Australia; Guillermo Carbayo Lozano, Department of Neurosurgery, Hospital of Cruces, Bilbao, Spain; Marco Carbonara, Neuro ICU, Fondazione IRCCS Cà Granda Ospedale Maggiore Policlinico, Milan, Italy; Simona Cavallo, Department of Anesthesia and ICU, AOU Città della Salute e della Scienza di Torino—Orthopedic and Trauma Center, Torino, Italy; Giorgio Chevallard, NeuroIntensive Care, Niguarda Hospital, Milan, Italy; Arturo Chieregato, NeuroIntensive Care, Niguarda Hospital, Milan, Italy; Giuseppe Citerio, School of Medicine and Surgery, Università Milano Bicocca, Milano, Italy, and NeuroIntensive Care Unit, Department Neuroscience, IRCCS Fondazione San Gerardo dei Tintori, Monza, Italy; Hans Clusmann, Department of Neurosurgery, Medical Faculty RWTH Aachen University, Aachen, Germany; Mark Coburn, Department of Anesthesiology and Intensive Care Medicine, University Hospital Bonn, Bonn, Germany; Jonathan Coles, Department of Anesthesia and Neurointensive Care, Cambridge University Hospital NHS Foundation Trust, Cambridge, U.K.; Jamie D. Cooper, School of Public Health and PM, Monash University and the Alfred Hospital, Melbourne, Victoria, Australia; Marta Correia, Radiology/MRI department, MRC Cognition and Brain Sciences Unit, Cambridge, U.K.; Amra Čović, Institute of Medical Psychology and Medical Sociology, Universitätsmedizin Göttingen, Göttingen, Germany; Nicola Curry, Oxford University Hospitals NHS Trust, Oxford, UK; Endre Czeiter, Department of Neurosurgery, Medical School, University of Pécs, Hungary and Neurotrauma Research Group, János Szentágothai Research Center, University of Pécs, Hungary; Marek Czosnyka, Brain Physics Lab, Division of Neurosurgery, Department of Clinical Neurosciences, University of Cambridge, Addenbrooke's Hospital, Cambridge, U.K.; Claire Dahyot Fizelier, Intensive Care Unit, CHU Poitiers, Potiers, France; Paul Dark, University of Manchester NIHR Biomedical Research Center, Critical Care Directorate, Salford Royal Hospital NHS Foundation Trust, Salford, U.K.; Helen Dawes, Movement Science Group, Faculty of Health and Life Sciences, Oxford Brookes University, Oxford, U.K.; Véronique De Keyser, Department of Neurosurgery, Antwerp University Hospital, Edegem, Belgium; Vincent Degos, Anesthesie-Réanimation, Assistance Publique—Hopitaux de Paris, Paris, France; Francesco Della Corte, Department of Anesthesia and Intensive Care, Maggiore Della Carità Hospital, Novara, Italy; Hugo den Boogert, Department of Neurosurgery, Radboud University Medical Center, Nijmegen, the Netherlands; Bart Depreitere, Department of Neurosurgery, University Hospitals Leuven, Leuven, Belgium; Đula Đilvesi, Department of Neurosurgery, Clinical Center of Vojvodina, Faculty of Medicine, University of Novi Sad, Novi Sad, Serbia; Abhishek Dixit, Division of Anesthesia, University of Cambridge, Addenbrooke's Hospital, Cambridge, U.K.; Emma Donoghue, Australian and New Zealand Intensive Care Research Center, Department of Epidemiology and Preventive Medicine, School of Public Health and Preventive Medicine, Monash University, Melbourne, Australia; Jens Dreier, Center for Stroke Research Berlin, Charité—Universitätsmedizin Berlin, corporate member of Freie Universität Berlin, Humboldt-Universität zu Berlin, and Berlin Institute of Health, Berlin, Germany; Guy Loup Dulière, Intensive Care Unit, CHR Citadelle, Liège, Belgium; Ari Ercole, Division of Anesthesia, University of Cambridge, Addenbrooke's Hospital, Cambridge, U.K.; Patrick Esser, Movement Science Group, Faculty of Health and Life Sciences, Oxford Brookes University, Oxford, U.K.; Erzsébet Ezer, Department of Anesthesiology and Intensive Therapy, University of Pécs, Pécs, Hungary; Martin Fabricius, Departments of Neurology, Clinical Neurophysiology and Neuroanesthesiology, Region Hovedstaden Rigshospitalet, Copenhagen, Denmark; Valery L. Feigin, National Institute for Stroke and Applied Neurosciences, Faculty of Health and Environmental Studies, Auckland University of Technology, Auckland, New Zealand; Kelly Foks, Department of Neurology, Erasmus MC, Rotterdam, the Netherlands; Shirin Frisvold, Department of Anesthesiology and Intensive care, University Hospital Northern Norway, Tromso, Norway; Alex Furmanov, Department of Neurosurgery, Hadassah-Hebrew University Medical Center, Jerusalem, Israel; Pablo Gagliardo, Fundación Instituto Valenciano de Neurorrehabilitación (FIVAN), Valencia, Spain; Damien Galanaud, Anesthesie-Réanimation, Assistance Publique—Hopitaux de Paris, Paris, France; Dashiell Gantner, ANZIC Research Center, Monash University, Department of Epidemiology and Preventive Medicine, Melbourne, Victoria, Australia; Guoyi Gao, Department of Neurosurgery, Shanghai Renji Hospital, Shanghai Jiaotong University/School of Medicine, Shanghai, China; Pradeep George, Karolinska Institutet, INCF International Neuroinformatics Coordinating Facility, Stockholm, Sweden; Alexandre Ghuysen, Emergency Department, CHU, Liège, Belgium; Lelde Giga, Neurosurgery Clinic, Pauls Stradins Clinical University Hospital, Riga, Latvia; Ben Glocker, Department of Computing, Imperial College London, London, U.K.; Jagoš Golubovic, Department of Neurosurgery, Clinical Center of Vojvodina, Faculty of Medicine, University of Novi Sad, Novi Sad, Serbia; Pedro A. Gomez, Department of Neurosurgery, Hospital Universitario 12 de Octubre, Madrid, Spain; Johannes Gratz, Department of Anesthesia, Critical Care and Pain Medicine, Medical University of Vienna, Austria; Benjamin Gravesteijn, Department of Public Health, Erasmus Medical Center-University Medical Center, Rotterdam, the Netherlands; Francesca Grossi, Department of Anesthesia and Intensive Care, Maggiore Della Carità Hospital, Novara, Italy; Russell L. Gruen, College of Health and Medicine, Australian National University, Canberra, Australia; Deepak Gupta, Department of Neurosurgery, Neurosciences Center and JPN Apex Trauma Center, All India Institute of Medical Sciences, New Delhi-110029, India; Juanita A. Haagsma, Department of Public Health, Erasmus Medical Center-University Medical Center, Rotterdam, the Netherlands; Iain Haitsma, Department of Neurosurgery, Erasmus MC, Rotterdam, the Netherlands; Raimund Helbok, Department of Neurology, Neurological Intensive Care Unit, Medical University of Innsbruck, Innsbruck, Austria; Eirik Helseth, Department of Neurosurgery, Oslo University Hospital, Oslo, Norway; Lindsay Horton, Division of Psychology, University of Stirling, Stirling, U.K.; Jilske Huijben, Department of Public Health, Erasmus Medical Center-University Medical Center, Rotterdam, the Netherlands; Peter J. Hutchinson, Division of Neurosurgery, Department of Clinical Neurosciences, Addenbrooke's Hospital and University of Cambridge, Cambridge, U.K.; Bram Jacobs, Department of Neurology, University of Groningen, University Medical Center Groningen, Groningen, the Netherlands; Stefan Jankowski, Neurointensive Care, Sheffield Teaching Hospitals NHS Foundation Trust, Sheffield, U.K.; Mike Jarrett, Quesgen Systems Inc., Burlingame, California, USA; Ji yao Jiang, Karolinska Institutet, INCF International Neuroinformatics Coordinating Facility, Stockholm, Sweden; Faye Johnson, Salford Royal Hospital NHS Foundation Trust Acute Research Delivery Team, Salford, U.K.; Kelly Jones, National Institute for Stroke and Applied Neurosciences, Faculty of Health and Environmental Studies, Auckland University of Technology, Auckland, New Zealand; Mladen Karan, Department of Neurosurgery, Clinical Center of Vojvodina, Faculty of Medicine, University of Novi Sad, Novi Sad, Serbia Angelos G. Kolias, Division of Neurosurgery, Department of Clinical Neurosciences, Addenbrooke's Hospital and University of Cambridge, Cambridge, U.K.; Erwin Kompanje, Department of Intensive Care and Department of Ethics and Philosophy of Medicine, Erasmus Medical Center, Rotterdam, the Netherlands; Daniel Kondziella, Departments of Neurology, Clinical Neurophysiology and Neuroanesthesiology, Region Hovedstaden Rigshospitalet, Copenhagen, Denmark; Evgenios Kornaropoulos, Division of Anesthesia, University of Cambridge, Addenbrooke's Hospital, Cambridge, U.K.; Lars Owe Koskinen, Department of Clinical Neuroscience, Neurosurgery, Umeå University, Umeå, Sweden; Noémi Kovács, Hungarian Brain Research Program-Grant No. KTIA_13_NAP-A-II/8, University of Pécs, Pécs, Hungary; Ana Kowark77, Department of Anesthesiology, University Hospital of Aachen, Aachen, Germany; Alfonso Lagares, Department of Neurosurgery, Hospital Universitario 12 de Octubre, Madrid, Spain; Linda Lanyon, Karolinska Institutet, INCF International Neuroinformatics Coordinating Facility, Stockholm, Sweden; Steven Laureys, Cyclotron Research Center, University of Liège, Liège, Belgium; Fiona Lecky, Center for Urgent and Emergency Care Research (CURE), Health Services Research Section, School of Health and Related Research (ScHARR), University of Sheffield, Sheffield, U.K., and Emergency Department, Salford Royal Hospital, Salford U.K.; Didier Ledoux, Cyclotron Research Center, University of Liège, Liège, Belgium; Rolf Lefering, Institute of Research in Operative Medicine (IFOM), Witten/Herdecke University, Cologne, Germany; Valerie Legrand, VP Global Project Management CNS, ICON, Paris, France; Aurelie Lejeune, Department of Anesthesiology-Intensive Care, Lille University Hospital, Lille, France; Leon Levi, Department of Neurosurgery, Rambam Medical Center, Haifa, Israel; Roger Lightfoot, Department of Anesthesiology and Intensive Care, University Hospitals Southhampton NHS Trust, Southhampton, U.K.; Hester Lingsma, Department of Public Health, Erasmus Medical Center-University Medical Center, Rotterdam, the Netherlands; Andrew I.R. Maas, Co-Lead Investigator, Department of Neurosurgery, Antwerp University Hospital, Edegem, Belgium, and Department of Translational Neuroscience, Faculty of Medicine and Health Science, University of Antwerp, Antwerp, Belgium; Ana M. Castaño León, Department of Neurosurgery, Hospital Universitario 12 de Octubre, Madrid, Spain; Marc Maegele, Cologne-Merheim Medical Center (CMMC), Department of Traumatology, Orthopedic Surgery and Sportmedicine, Witten/Herdecke University, Cologne, Germany; Marek Majdan, Department of Public Health, Faculty of Health Sciences and Social Work, Trnava University, Trnava, Slovakia; Alex Manara, Intensive Care Unit, Southmead Hospital, Bristol, Bristol, U.K.; Geoffrey Manley, Department of Neurological Surgery, University of California, San Francisco, California, USA; Costanza Martino, Department of Anesthesia and Intensive Care, M. Bufalini Hospital, Cesena, Italy; Hugues Maréchal, Intensive Care Unit, CHR Citadelle, Liège, Belgium; Julia Mattern, Department of Neurosurgery, University Hospital Heidelberg, Heidelberg, Germany; Catherine McMahon, Department of Neurosurgery, the Walton Center NHS Foundation Trust, Liverpool, U.K.; Béla Melegh, Department of Medical Genetics, University of Pécs, Pécs, Hungary; David Menon, Co-Lead Investigator, Division of Anesthesia, University of Cambridge, Addenbrooke's Hospital, Cambridge, U.K.; Tomas Menovsky, Department of Neurosurgery, Antwerp University Hospital, Edegem, Belgium and Department of Translational Neuroscience, Faculty of Medicine and Health Science, University of Antwerp, Antwerp, Belgium; Ana Mikolic, Department of Public Health, Erasmus Medical Center-University Medical Center, Rotterdam, the Netherlandsl Benoit Misset, Cyclotron Research Center, University of Liège, Liège, Belgium; Visakh Muraleedharan, Karolinska Institutet, INCF International Neuroinformatics Coordinating Facility, Stockholm, Sweden; Lynnette Murray, ANZIC Research Center, Monash University, Department of Epidemiology and Preventive Medicine, Melbourne, Victoria, Australia; Ancuta Negru, Department of Neurosurgery, Emergency County Hospital Timisoara, Timisoara, Romania; David Nelson, Department of Physiology and Pharmacology, Section of Perioperative Medicine and Intensive Care, Karolinska Institutet, Stockholm, Sweden; Virginia Newcombe, Division of Anesthesia, University of Cambridge, Addenbrooke's Hospital, Cambridge, U.K.; Daan Nieboer, Department of Public Health, Erasmus Medical Center-University Medical Center, Rotterdam, the Netherlands; József Nyirádi, János Szentágothai Research Center, University of Pécs, Pécs, Hungary; Otesile Olubukola, Center for Urgent and Emergency Care Research (CURE), Health Services Research Section, School of Health and Related Research (ScHARR), University of Sheffield, Sheffield, U.K.; Matej Oresic, School of Medical Sciences, Örebro University, Örebro, Sweden; Fabrizio Ortolano, Neuro ICU, Fondazione IRCCS Cà Granda Ospedale Maggiore Policlinico, Milan, Italy; Aarno Palotie, Institute for Molecular Medicine Finland, University of Helsinki, Helsinki, Finland, Analytic and Translational Genetics Unit, Department of Medicine, Psychiatric and Neurodevelopmental Genetics Unit, Department of Psychiatry, Department of Neurology, Massachusetts General Hospital, Boston, MA, USA, and Program in Medical and Population Genetics, the Stanley Center for Psychiatric Research, the Broad Institute of MIT and Harvard, Cambridge, MA, USA; Paul M. Parizel, Department of Radiology, University of Antwerp, Edegem, Belgium; Jean François Payen, Department of Anesthesiology and Intensive Care, University Hospital of Grenoble, Grenoble, France; Natascha Perera, International Projects Management, ARTTIC, Munich, Germany; Vincent Perlbarg, Anesthesie-Réanimation, Assistance Publique—Hopitaux de Paris, Paris, France; Paolo Persona, Department of Anesthesia and Intensive Care, Azienda Ospedaliera Università di Padova, Padova, Italy; Wilco Peul, Department of Neurosurgery, Leiden University Medical Center, Leiden, the Netherlands and Department of Neurosurgery, Medical Center Haaglanden, the Hague, the Netherlands; Anna Piippo-Karjalainen, Department of Neurosurgery, Helsinki University Central Hospital, Helsinki, Finland; Matti Pirinen, Institute for Molecular Medicine Finland, University of Helsinki, Helsinki, Finland; Dana Pisica, Department of Public Health, Erasmus Medical Center-University Medical Center, Rotterdam, the Netherlands; Horia Ples, Department of Neurosurgery, Emergency County Hospital Timisoara, Timisoara, Romania; Suzanne Polinder, Department of Public Health, Erasmus Medical Center-University Medical Center, Rotterdam, the Netherlands; Inigo Pomposo, Department of Neurosurgery, Hospital of Cruces, Bilbao, Spain; Jussi P. Posti, Division of Clinical Neurosciences, Department of Neurosurgery and Turku Brain Injury Center, Turku University Hospital and University of Turku, Turku, Finland; Louis Puybasset, Department of Anesthesiology and Critical Care, Pitié -Salpêtrière Teaching Hospital, Assistance Publique, Hôpitaux de Paris and University Pierre et Marie Curie, Paris, France; Andreea Radoi, Neurotraumatology and Neurosurgery Research Unit (UNINN), Vall d'Hebron Research Institute, Barcelona, Spain; Arminas Ragauskas, Department of Neurosurgery, Kaunas University of technology and Vilnius University, Vilnius, Lithuania; Rahul Raj, Department of Neurosurgery, Helsinki University Central Hospital, Helsinki, Finland; Malinka Rambadagalla, Department of Neurosurgery, Rezekne Hospital, Latvia; Isabel Retel Helmrich, Department of Public Health, Erasmus Medical Center-University Medical Center, Rotterdam, the Netherlands; Jonathan Rhodes, Department of Anesthesia, Critical Care and Pain Medicine NHS Lothian and University of Edinburg, Edinburgh, U.K.; Sylvia Richardson, Director, MRC Biostatistics Unit, Cambridge Institute of Public Health, Cambridge, U.K.; Sophie Richter, Division of Anesthesia, University of Cambridge, Addenbrooke's Hospital, Cambridge, U.K.; Samuli Ripatti, Institute for Molecular Medicine Finland, University of Helsinki, Helsinki, Finland; Saulius Rocka, Department of Neurosurgery, Kaunas University of technology and Vilnius University, Vilnius, Lithuania; Cecilie Roe, Department of Physical Medicine and Rehabilitation, Oslo University Hospital/University of Oslo, Oslo, Norway; Olav Roise, Division of Orthopedics, Oslo University Hospital, Oslo, Norway, and Institute of Clinical Medicine, Faculty of Medicine, University of Oslo, Oslo, Norway; Jonathan Rosand, Broad Institute, Cambridge MA Harvard Medical School, Boston MA, Massachusetts General Hospital, Boston MA, USA; Jeffrey V. Rosenfeld, National Trauma Research Institute, the Alfred Hospital, Monash University, Melbourne, Victoria, Australia; Christina Rosenlund, Department of Neurosurgery, Odense University Hospital, Odense, Denmark; Guy Rosenthal, Department of Neurosurgery, Hadassah-hebrew University Medical center, Jerusalem, Israel; Rolf Rossaint, Department of Anesthesiology, University Hospital of Aachen, Aachen, Germany; Sandra Rossi, Department of Anesthesia and Intensive Care, Azienda Ospedaliera Università di Padova, Padova, Italy; Daniel Rueckert, Department of Computing, Imperial College London, London, U.K.; Martin Rusnák, International Neurotrauma Research Organisation, Vienna, Austria; Juan Sahuquillo, Neurotraumatology and Neurosurgery Research Unit (UNINN), Vall d'Hebron Research Institute, Barcelona, Spain Oliver Sakowitz, Department of Neurosurgery, University Hospital Heidelberg, Heidelberg, Germany and Klinik für Neurochirurgie, Klinikum Ludwigsburg, Ludwigsburg, Germany; Renan Sanchez Porras, Klinik für Neurochirurgie, Klinikum Ludwigsburg, Ludwigsburg, Germany; Janos Sandor, Division of Biostatistics and Epidemiology, Department of Preventive Medicine, University of Debrecen, Debrecen, Hungary; Nadine Schäfer, Institute of Research in Operative Medicine (IFOM), Witten/Herdecke University, Cologne, Germany; Silke Schmidt, Department Health and Prevention, University Greifswald, Greifswald, Germany; Herbert Schoechl, Department of Anesthesiology and Intensive Care, AUVA Trauma Hospital, Salzburg, Austria; Guus Schoonman, Department of Neurology, Elisabeth-TweeSteden Ziekenhuis, Tilburg, the Netherlands; Rico Frederik Schou, Department of Neuroanesthesia and Neurointensive Care, Odense University Hospital, Odense, Denmark; Elisabeth Schwendenwein, Trauma Surgery, Medical University Vienna, Vienna, Austria; Charlie Sewalt, Department of Public Health, Erasmus Medical Center-University Medical Center, Rotterdam, the Netherlands; Ranjit D. Singh, Department of Neurosurgery, Leiden University Medical Center, Leiden, the Netherlands and Department of Neurosurgery, Medical Center Haaglanden, the Hague, the Netherlands; Toril Skandsen, Department of Neuromedicine and Movement Science, Norwegian University of Science and Technology, NTNU, Trondheim, Norway, and Department of Physical Medicine and Rehabilitation, St. Olavs Hospital, Trondheim University Hospital, Trondheim, Norway; Peter Smielewski, Brain Physics Lab, Division of Neurosurgery, Dept of Clinical Neurosciences, University of Cambridge, Addenbrooke's Hospital, Cambridge, U.K.; Abayomi Sorinola, Department of Neurosurgery, University of Pécs, Pécs, Hungary; Emmanuel Stamatakis, Division of Anesthesia, University of Cambridge, Addenbrooke's Hospital, Cambridge, U.K.; Simon Stanworth, Oxford University Hospitals NHS Trust, Oxford, U.K.; Robert Stevens, Division of Neuroscience Critical Care, John Hopkins University School of Medicine, Baltimore, USA; William Stewart, Department of Neuropathology, Queen Elizabeth University Hospital and University of Glasgow, Glasgow, U.K.; Ewout W. Steyerberg, Department of Public Health, Erasmus Medical Center-University Medical Center, Rotterdam, the Netherlands and Department of Biomedical Data Sciences, Leiden University Medical Center, Leiden, the Netherlands; Nino Stocchetti, Department of Pathophysiology and Transplantation, Milan University, and Neuroscience ICU, Fondazione IRCCS Cà Granda Ospedale Maggiore Policlinico, Milano, Italy; Nina Sundström, Department of Radiation Sciences, Biomedical Engineering, Umeå University, Umeå, Sweden; Riikka Takala, Perioperative Services, Intensive Care Medicine and Pain Management, Turku University Hospital and University of Turku, Turku, Finland; Viktória Tamás, Department of Neurosurgery, University of Pécs, Pécs, Hungary; Tomas Tamosuitis, Department of Neurosurgery, Kaunas University of Health Sciences, Kaunas, Lithuania; Mark Steven Taylor, Department of Public Health, Faculty of Health Sciences and Social Work, Trnava University, Trnava, Slovakia; Aurore Thibaut, Cyclotron Research Center, University of Liège, Liège, Belgium; Braden Te Ao, National Institute for Stroke and Applied Neurosciences, Faculty of Health and Environmental Studies, Auckland University of Technology, Auckland, New Zealand; Olli Tenovuo, Division of Clinical Neurosciences, Department of Neurosurgery and Turku Brain Injury Center, Turku University Hospital and University of Turku, Turku, Finland; Alice Theadom, National Institute for Stroke and Applied Neurosciences, Faculty of Health and Environmental Studies, Auckland University of Technology, Auckland, New Zealand; Matt Thomas, National Institute for Stroke and Applied Neurosciences, Faculty of Health and Environmental Studies, Auckland University of Technology, Auckland, New Zealand; Dick Tibboel, Intensive Care and Department of Pediatric Surgery, Erasmus Medical Center, Sophia Children's Hospital, Rotterdam, the Netherlands; Marjolein Timmers, Department of Intensive Care and Department of Ethics and Philosophy of Medicine, Erasmus Medical Center, Rotterdam, the Netherlands; Christos Tolias, Department of Neurosurgery, Kings College London, London, U.K.; Tony Trapani, ANZIC Research Center, Monash University, Department of Epidemiology and Preventive Medicine, Melbourne, Victoria, Australia; Cristina Maria Tudora, Department of Neurosurgery, Emergency County Hospital Timisoara, Timisoara, Romania; Andreas Unterberg, Department of Neurosurgery, University Hospital Heidelberg, Heidelberg, Germany; Peter Vajkoczy, Neurologie, Neurochirurgie und Psychiatrie, Charité – Universitätsmedizin Berlin, Berlin, Germany; Shirley Vallance, ANZIC Research Center, Monash University, Department of Epidemiology and Preventive Medicine, Melbourne, Victoria, Australia; Egils Valeinis, Neurosurgery Clinic, Pauls Stradins Clinical University Hospital, Riga, Latvia; Zoltán Vámos, Department of Anesthesiology and Intensive Therapy, University of Pécs, Pécs, Hungary; Mathieu van der Jagt, Department of Intensive Care Adults, Erasmus MC—University Medical Center Rotterdam, Rotterdam, the Netherlands; Gregory Van der Steen, Department of Neurosurgery, Antwerp University Hospital, Edegem, Belgium; Joukje van der Naalt, Department of Neurology, University of Groningen, University Medical Center Groningen, Groningen, the Netherlands; Jeroen T.J.M. van Dijck, Department of Neurosurgery, Leiden University Medical Center, Leiden, the Netherlands and Department of Neurosurgery, Medical Center Haaglanden, the Hague, the Netherlands; Inge A. M. van Erp, Department of Neurosurgery, Leiden University Medical Center, Leiden, the Netherlands and Department of Neurosurgery, Medical Center Haaglanden, the Hague, the Netherlands; Thomas A. van Essen, Department of Neurosurgery, Leiden University Medical Center, Leiden, the Netherlands and Department of Neurosurgery, Medical Center Haaglanden, the Hague, the Netherlands; Wim Van Hecke, icoMetrix NV, Leuven, Belgium; Caroline van Heugten, Movement Science Group, Faculty of Health and Life Sciences, Oxford Brookes University, Oxford, U.K.; Ernest van Veen, Department of Public Health, Erasmus Medical Center-University Medical Center, Rotterdam, the Netherlands; Thijs Vande Vyvere, Radiology Department, Antwerp University Hospital, Edegem, Belgium; Roel P. J. van Wijk, Department of Neurosurgery, Leiden University Medical Center, Leiden, the Netherlands and Dept. of Neurosurgery, Medical Center Haaglanden, the Hague, the Netherlands; Alessia Vargiolu, NeuroIntensive Care Unit, Department Neuroscience, IRCCS Fondazione San Gerardo dei Tintori, Monza, Italy; Emmanuel Vega, Department of Anesthesiology-Intensive Care, Lille University Hospital, Lille, France; Kimberley Velt, Department of Public Health, Erasmus Medical Center-University Medical Center, Rotterdam, the Netherlands; Jan Verheyden, icoMetrix NV, Leuven, Belgium; Paul M. Vespa, Director of Neurocritical Care, University of California, Los Angeles, USA; Anne Vik, Director of Neurocritical Care, University of California, Los Angeles, USA and Department of Neurosurgery, St.Olavs Hospital, Trondheim University Hospital, Trondheim, Norway; Rimantas Vilcinis, Department of Neurosurgery, Kaunas University of Health Sciences, Kaunas, Lithuania; Victor Volovici, Department of Neurosurgery, Erasmus MC, Rotterdam, the Netherlands; Nicole von Steinbüche, icoMetrix NV, Leuven, Belgium; Daphne Voormolen, Department of Public Health, Erasmus Medical Center-University Medical Center, Rotterdam, the Netherlands; Petar Vulekovic, Department of Neurosurgery, Clinical Center of Vojvodina, Faculty of Medicine, University of Novi Sad, Novi Sad, Serbia; Kevin K.W. Wang, Department of Emergency Medicine, University of Florida, Gainesville, Florida, USA; Daniel Whitehouse, Division of Anesthesia, University of Cambridge, Addenbrooke's Hospital, Cambridge, U.K.; Eveline Wiegers, Department of Public Health, Erasmus Medical Center-University Medical Center, Rotterdam, the Netherlands; Guy Williams, Division of Anesthesia, University of Cambridge, Addenbrooke's Hospital, Cambridge, U.K.; Lindsay Wilson, Division of Psychology, University of Stirling, Stirling, U.K.; Stefan Winzeck, Division of Anesthesia, University of Cambridge, Addenbrooke's Hospital, Cambridge, U.K.; Stefan Wolf, Department of Neurosurgery, Charité—Universitätsmedizin Berlin, corporate member of Freie Universität Berlin, Humboldt-Universität zu Berlin, and Berlin Institute of Health, Berlin, Germany; Zhihui Yang, Broad Institute, Cambridge MA Harvard Medical School, Boston MA, Massachusetts General Hospital, Boston MA, USA; Peter Ylén, VTT Technical Research Center, Tampere, Finland; Alexander Younsi, Department of Neurosurgery, University Hospital Heidelberg, Heidelberg, Germany; Frederick A. Zeiler, Division of Anesthesia, University of Cambridge, Addenbrooke's Hospital, Cambridge, U.K. and Section of Neurosurgery, Department of Surgery, Rady Faculty of Health Sciences, University of Manitoba, Winnipeg, MB, Canada; Veronika Zelinkova, Department of Public Health, Faculty of Health Sciences and Social Work, Trnava University, Trnava, Slovakia; Agate Ziverte, Neurosurgery clinic, Pauls Stradins Clinical University Hospital, Riga, Latvia; Tommaso Zoerle, Neuro ICU, Fondazione IRCCS Cà Granda Ospedale Maggiore Policlinico, Milan, Italy.
