# Supplementary material for: Therapy Intensity Level Scale for Traumatic Brain Injury: Clinimetric Assessment on Neuro-Monitored Patients Across 52 European Intensive Care Units
Source: J Neurotrauma. 2024 Apr 4;41(7-8):887–909. doi: 10.1089/neu.2023.0377 (PMC11005383; doi:10.1089/neu.2023.0377)
Supplement: Supplemental data [file Suppl_FigS1.pdf]

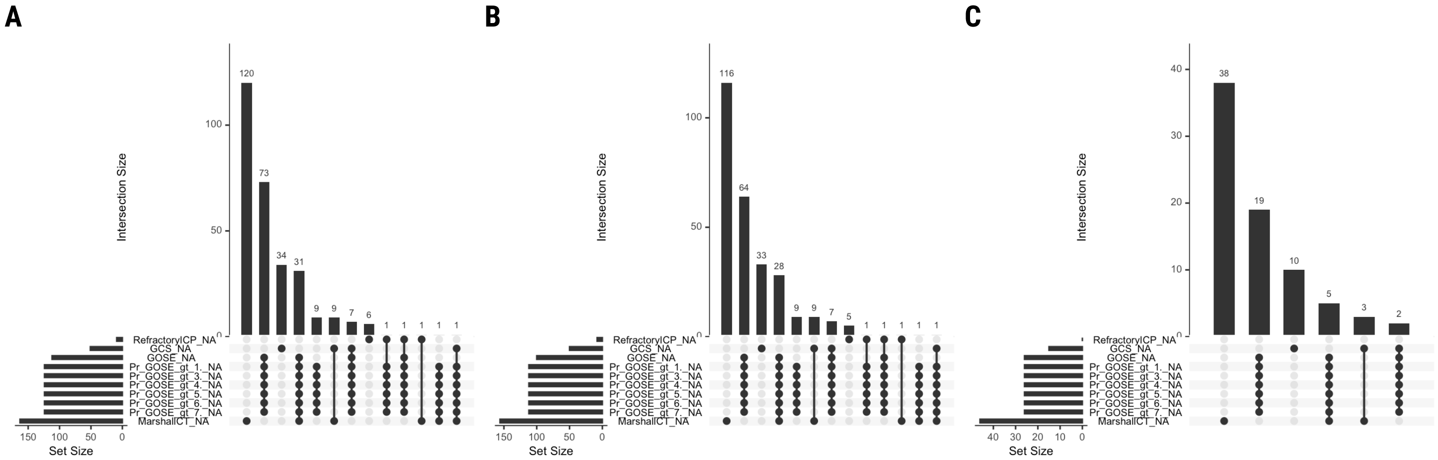

Supplementary Figure S1. Missingness of static study measures. Abbreviations: GCS_NA=indicator variable representing cases with missing values for Glasgow Coma Scale at ICU admission, GOSE_NA=indicator variable representing cases with missing values for Glasgow Outcome Scale–Extended at six months post-injury, ICU=intensive care unit, MarshallCT_NA=indicator variable representing cases with missing values for Marshall CT classification at ICU admission**,** Pr_GOSE_gt_•._NA=indicator variable representing cases with missing values for "probability of GOSE greater than • at six months post-injury" as previously calculated from the first 24 hours of admission,[27] RefractoryICP_NA=indicator variable representing cases with missing values for refractory intracranial hypertension during ICU stay. A separate missingness UpSet plot is shown for the (**A**) TIL validation population (*n*=873), (**B**) TIL-ICP_{EH} sub-population (*n*=837), and (**C**) TIL-ICP_{HR} sub-population (*n*=259), as defined in Figure 1.
