# Supplementary material for: Therapy Intensity Level Scale for Traumatic Brain Injury: Clinimetric Assessment on Neuro-Monitored Patients Across 52 European Intensive Care Units
Source: J Neurotrauma. 2024 Apr 4;41(7-8):887–909. doi: 10.1089/neu.2023.0377 (PMC11005383; doi:10.1089/neu.2023.0377)
Supplement: Supplemental data [file Suppl_FigS2.pdf]

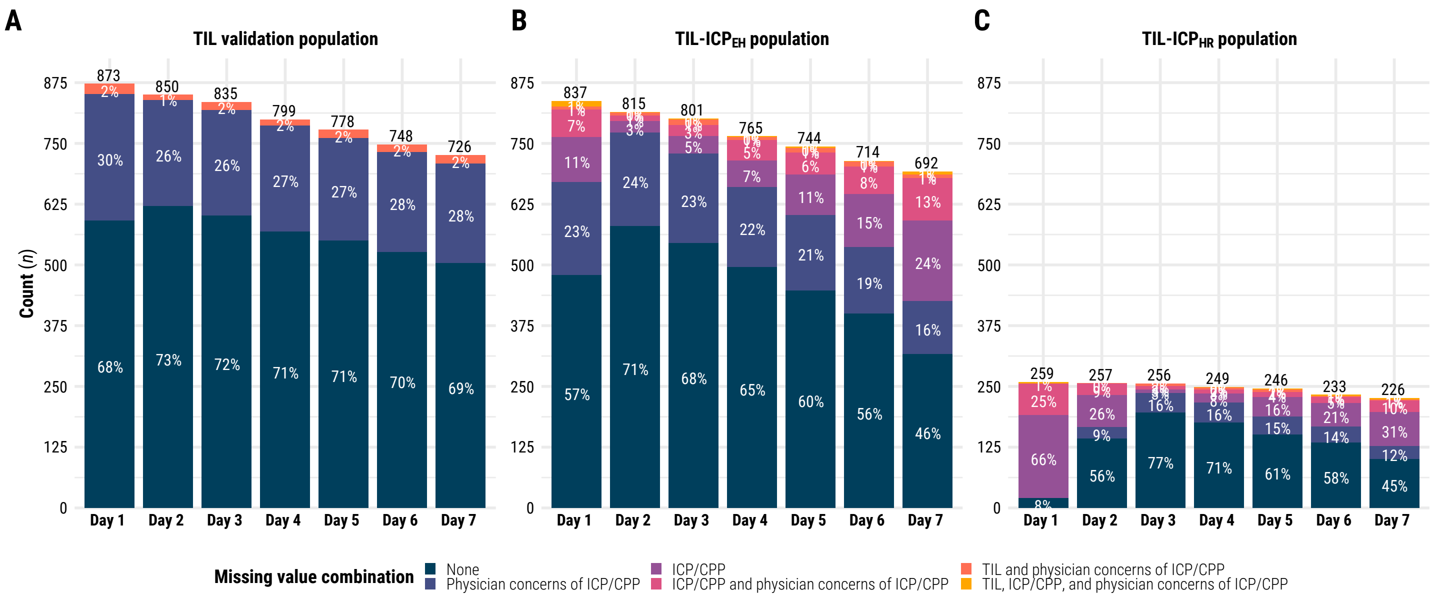

Supplementary Figure S2. Study populations remaining in ICU over first week and missingness of longitudinal study measures. Abbreviations: CPP=cerebral perfusion pressure, EH=end-hour, HR=high-resolution, ICP=intracranial pressure, ICU=intensive care unit, TIL=Therapy Intensity Level scale,[8,9] TIL-ICP$_{EH}$=end-hour ICP sub-population, TIL-ICP$_{HR}$=high-resolution ICP sub-population. The values above each stacked bar plot represent the number of patients remaining at each day of ICU stay (i.e., expected cases), excluding those who have already had a decision to withdraw life-sustaining therapies. The value in each component of the stacked bar plot represents the percent of expected cases per each day of ICU stay that falls into the corresponding missing value combination of that colour. A separate missingness stacked bar plot is shown for the (**A**) TIL validation population (*n*=873), (**B**) TIL-ICP$_{EH}$ sub-population (*n*=837), and (**C**) TIL-ICP$_{HR}$ sub-population (*n*=259), as defined in Figure 1.
