# Supplementary material for: Therapy Intensity Level Scale for Traumatic Brain Injury: Clinimetric Assessment on Neuro-Monitored Patients Across 52 European Intensive Care Units
Source: J Neurotrauma. 2024 Apr 4;41(7-8):887–909. doi: 10.1089/neu.2023.0377 (PMC11005383; doi:10.1089/neu.2023.0377)
Supplement: Supplemental data [file Suppl_TableS1.docx]

**Supplementary Table S1. Significant characteristic differences associated with longitudinal variable missingness.**

| **Validation**  **population** | **Day in**  **ICU** | **Variable** | **Nonmissing**  **count** | **Missing**  **count** | **Characteristic** | **Value** | **Nonmissing**  **group** | **Missing**  **group** | **p-value^†^** |
| --- | --- | --- | --- | --- | --- | --- | --- | --- | --- |
| TIL | Day 1 | Physician concerns of ICP/CPP | 592 | 281 | Centre distribution* | | 46 | 35 | 0.000 |
|  |  |  | 504 | 245 | Baseline functional prognosis | Pr(GOSE>4) | 37.2 (19.3–56.9) | 45.7 (22.2–64.6) | 0.004 |
|  |  |  | 504 | 245 | Baseline functional prognosis | Pr(GOSE>5) | 19.6 (10.5–35.9) | 23.3 (9.5–39.0) | 0.039 |
|  |  |  | 504 | 245 | Baseline functional prognosis | Pr(GOSE>6) | 11.4 (5.7–19.6) | 14.0 (6.3–21.9) | 0.013 |
|  |  |  | 504 | 245 | Baseline functional prognosis | Pr(GOSE>7) | 4.4 (2.2–8.7) | 5.8 (2.5–10.2) | 0.005 |
|  |  |  | 592 | 281 | TILmax |  | 10.0 (7.0–15.0) | 10.0 (6.0–14.0) | 0.012 |
|  |  |  | 592 | 281 | TILmedian |  | 6.0 (3.0–10.0) | 5.0 (3.0–9.0) | 0.017 |
|  |  |  | 592 | 260 | TIL24 |  | 8.0 (5.0–12.0) | 6.0 (3.0–11.0) | 0.002 |
| TIL | Day 2 | TIL24 | 839 | 11 | Centre distribution* | | 51 | 11 | 0.000 |
|  |  |  | 730 | 8 | Baseline functional prognosis | Pr(GOSE>6) | 12.4 (6.1–20.9) | 6.0 (3.3–9.7) | 0.021 |
| TIL | Day 2 | Physician concerns of ICP/CPP | 621 | 229 | Centre distribution* | | 45 | 37 | 0.000 |
|  |  |  | 539 | 199 | Baseline functional prognosis | Pr(GOSE>4) | 38.2 (21.1–57.7) | 46.0 (20.7–63.1) | 0.038 |
|  |  |  | 539 | 199 | Baseline functional prognosis | Pr(GOSE>6) | 11.9 (6.0–19.8) | 13.8 (6.3–22.4) | 0.033 |
|  |  |  | 539 | 199 | Baseline functional prognosis | Pr(GOSE>7) | 4.6 (2.2–8.8) | 5.5 (2.4–10.5) | 0.020 |
|  |  |  | 621 | 229 | TILmax |  | 10.0 (7.0–15.0) | 9.0 (5.0–14.0) | 0.004 |
|  |  |  | 621 | 229 | TILmedian |  | 6.0 (3.0–10.0) | 5.0 (3.0–9.0) | 0.009 |
|  |  |  | 621 | 218 | TIL24 |  | 6.0 (4.0–11.0) | 6.0 (4.0–9.0) | 0.007 |
| TIL | Day 3 | TIL24 | 715 | 14 | Six-month GOSE | 1 | 164 (23%) | 12 (86%) | 0.000 |
|  |  |  | 715 | 14 | Six-month GOSE | 2_or_3 | 180 (25%) | 1 (7%) | 0.000 |
|  |  |  | 715 |  | Six-month GOSE | 4 | 70 (10%) |  | 0.000 |
|  |  |  | 715 | 14 | Six-month GOSE | 5 | 121 (17%) | 1 (7%) | 0.000 |
|  |  |  | 715 |  | Six-month GOSE | 6 | 70 (10%) |  | 0.000 |
|  |  |  | 715 |  | Six-month GOSE | 7 | 54 (8%) |  | 0.000 |
|  |  |  | 715 |  | Six-month GOSE | 8 | 56 (8%) |  | 0.000 |
|  |  |  | 715 | 14 | Baseline functional prognosis | Pr(GOSE>1) | 85.5 (65.4–94.9) | 47.9 (24.0–69.0) | 0.003 |
|  |  |  | 715 | 14 | Baseline functional prognosis | Pr(GOSE>3) | 55.2 (30.7–76.1) | 18.4 (7.7–43.2) | 0.003 |
|  |  |  | 715 | 14 | Baseline functional prognosis | Pr(GOSE>4) | 40.6 (21.2–59.7) | 14.6 (5.0–30.7) | 0.007 |
|  |  |  | 715 | 14 | Baseline functional prognosis | Pr(GOSE>5) | 21.6 (10.9–36.9) | 7.6 (2.5–18.0) | 0.007 |
|  |  |  | 715 | 14 | Baseline functional prognosis | Pr(GOSE>6) | 12.5 (6.3–20.9) | 4.2 (1.7–8.0) | 0.021 |
|  |  |  | 715 | 14 | Baseline functional prognosis | Pr(GOSE>7) | 5.1 (2.4–9.2) | 1.2 (0.5–4.5) | 0.001 |
|  |  |  | 819 | 16 | TILmedian |  | 5.0 (3.0–9.0) | 9.0 (6.0–11.8) | 0.032 |
| TIL | Day 3 | Physician concerns of ICP/CPP | 602 | 233 | Centre distribution* | | 44 | 37 | 0.000 |
|  |  |  | 602 | 233 | TILmax |  | 10.0 (7.0–15.0) | 10.0 (5.0–14.0) | 0.021 |
|  |  |  | 602 | 217 | TIL24 |  | 6.0 (4.0–10.0) | 5.0 (3.0–8.0) | 0.001 |
| TIL | Day 4 | TIL24 | 642 | 10 | Marshall CT | 1 | 15 (2%) | 2 (20%) | 0.011 |
|  |  |  | 642 | 10 | Marshall CT | 2 | 249 (39%) | 3 (30%) | 0.011 |
|  |  |  | 642 | 10 | Marshall CT | 3 | 80 (12%) | 2 (20%) | 0.011 |
|  |  |  | 642 |  | Marshall CT | 4 | 14 (2%) |  | 0.011 |
|  |  |  | 642 | 10 | Marshall CT | 5_or_6 | 284 (44%) | 3 (30%) | 0.011 |
|  |  |  | 787 | 12 | TILmax |  | 10.0 (6.0–15.0) | 8.5 (4.5–11.0) | 0.048 |
| TIL | Day 4 | Physician concerns of ICP/CPP | 569 | 230 | Centre distribution* | | 45 | 36 | 0.000 |
|  |  |  | 493 | 200 | Baseline functional prognosis | Pr(GOSE>7) | 4.8 (2.5–8.8) | 5.7 (2.6–10.3) | 0.026 |
|  |  |  | 569 | 230 | TILmax |  | 10.0 (7.0–15.0) | 10.0 (5.2–14.0) | 0.004 |
|  |  |  | 569 | 230 | TILmedian |  | 6.0 (3.0–10.0) | 5.0 (3.0–8.0) | 0.001 |
|  |  |  | 569 | 218 | TIL24 |  | 6.0 (3.0–10.0) | 5.0 (3.0–8.0) | 0.002 |
| TIL | Day 5 | TIL24 | 663 | 12 | Six-month GOSE | 1 | 138 (21%) | 9 (75%) | 0.001 |
|  |  |  | 663 | 12 | Six-month GOSE | 2_or_3 | 176 (27%) | 2 (17%) | 0.001 |
|  |  |  | 663 |  | Six-month GOSE | 4 | 69 (10%) |  | 0.001 |
|  |  |  | 663 |  | Six-month GOSE | 5 | 119 (18%) |  | 0.001 |
|  |  |  | 663 |  | Six-month GOSE | 6 | 66 (10%) |  | 0.001 |
|  |  |  | 663 |  | Six-month GOSE | 7 | 49 (7%) |  | 0.001 |
|  |  |  | 663 | 12 | Six-month GOSE | 8 | 46 (7%) | 1 (8%) | 0.001 |
|  |  |  | 663 | 12 | Baseline functional prognosis | Pr(GOSE>1) | 85.7 (66.1–95.1) | 63.2 (38.3–72.6) | 0.022 |
|  |  |  | 663 | 12 | Baseline functional prognosis | Pr(GOSE>3) | 55.5 (32.3–76.1) | 29.8 (11.4–40.0) | 0.005 |
|  |  |  | 663 | 12 | Baseline functional prognosis | Pr(GOSE>4) | 41.0 (22.7–59.6) | 23.5 (7.2–29.8) | 0.011 |
|  |  |  | 663 | 12 | Baseline functional prognosis | Pr(GOSE>5) | 21.8 (11.2–37.0) | 11.6 (4.0–16.5) | 0.046 |
| TIL | Day 5 | Physician concerns of ICP/CPP | 551 | 227 | Centre distribution* | | 42 | 36 | 0.000 |
|  |  |  | 551 | 227 | TILmedian |  | 6.0 (3.8–10.0) | 5.0 (3.0–8.0) | 0.004 |
|  |  |  | 551 | 210 | TIL24 |  | 6.0 (3.0–10.0) | 5.0 (2.0–9.0) | 0.000 |
| TIL | Day 6 | TIL24 | 633 | 11 | Six-month GOSE | 1 | 127 (20%) | 8 (73%) | 0.003 |
|  |  |  | 633 | 11 | Six-month GOSE | 2_or_3 | 175 (28%) | 2 (18%) | 0.003 |
|  |  |  | 633 |  | Six-month GOSE | 4 | 68 (11%) |  | 0.003 |
|  |  |  | 633 |  | Six-month GOSE | 5 | 112 (18%) |  | 0.003 |
|  |  |  | 633 |  | Six-month GOSE | 6 | 64 (10%) |  | 0.003 |
|  |  |  | 633 |  | Six-month GOSE | 7 | 44 (7%) |  | 0.003 |
|  |  |  | 633 | 11 | Six-month GOSE | 8 | 43 (7%) | 1 (9%) | 0.003 |
|  |  |  | 633 | 11 | Baseline functional prognosis | Pr(GOSE>3) | 55.5 (33.4–75.6) | 28.5 (17.3–40.4) | 0.011 |
|  |  |  | 633 | 11 | Baseline functional prognosis | Pr(GOSE>4) | 41.1 (23.5–59.0) | 22.2 (13.5–29.8) | 0.021 |
|  |  |  | 733 | 15 | TILmax |  | 10.0 (7.0–15.0) | 8.0 (4.0–12.0) | 0.022 |
| TIL | Day 6 | Physician concerns of ICP/CPP | 527 | 221 | Centre distribution* | | 42 | 35 | 0.000 |
|  |  |  | 527 | 221 | TILmax |  | 11.0 (7.0–15.0) | 10.0 (6.0–14.0) | 0.005 |
|  |  |  | 527 | 221 | TILmedian |  | 6.0 (4.0–10.0) | 5.0 (3.0–8.0) | 0.000 |
|  |  |  | 527 | 206 | TIL24 |  | 5.0 (3.0–10.0) | 4.5 (2.0–8.0) | 0.003 |
| TIL | Day 7 | TIL24 | 709 | 17 | Centre distribution* | | 51 | 12 | 0.001 |
| TIL | Day 7 | Physician concerns of ICP/CPP | 504 | 222 | Centre distribution* | | 41 | 39 | 0.000 |
|  |  |  | 504 | 222 | TILmedian |  | 6.0 (4.0–10.0) | 5.0 (3.0–8.0) | 0.003 |
|  |  |  | 504 | 205 | TIL24 |  | 5.0 (2.0–9.0) | 4.0 (2.0–7.0) | 0.009 |
| TIL-ICPEH | Day 1 | TIL24 | 820 | 17 | TILmax |  | 10.0 (6.0–15.0) | 6.0 (4.0–10.0) | 0.012 |
|  |  |  | 820 | 17 | TILmedian |  | 5.5 (3.0–10.0) | 4.5 (2.0–6.0) | 0.047 |
| TIL-ICPEH | Day 1 | ICP/CPP | 677 | 160 | Centre distribution* | | 50 | 40 | 0.000 |
|  |  |  | 555 | 126 | Marshall CT | 1 | 11 (2%) | 5 (4%) | 0.013 |
|  |  |  | 555 | 126 | Marshall CT | 2 | 187 (34%) | 61 (48%) | 0.013 |
|  |  |  | 555 | 126 | Marshall CT | 3 | 75 (14%) | 14 (11%) | 0.013 |
|  |  |  | 555 | 126 | Marshall CT | 4 | 14 (3%) | 2 (2%) | 0.013 |
|  |  |  | 555 | 126 | Marshall CT | 5_or_6 | 268 (48%) | 44 (35%) | 0.013 |
|  |  |  | 593 | 132 | Baseline functional prognosis | Pr(GOSE>3) | 50.9 (27.3–73.6) | 63.2 (31.5–80.4) | 0.008 |
|  |  |  | 593 | 132 | Baseline functional prognosis | Pr(GOSE>4) | 37.0 (18.7–56.7) | 47.5 (22.7–65.0) | 0.008 |
|  |  |  | 593 | 132 | Baseline functional prognosis | Pr(GOSE>5) | 19.3 (9.5–33.7) | 27.7 (14.1–42.7) | 0.001 |
|  |  |  | 593 | 132 | Baseline functional prognosis | Pr(GOSE>6) | 11.4 (5.6–18.9) | 14.0 (7.4–23.8) | 0.004 |
|  |  |  | 593 | 132 | Baseline functional prognosis | Pr(GOSE>7) | 4.5 (2.2–8.9) | 5.9 (2.9–10.1) | 0.020 |
|  |  |  | 671 | 149 | TIL24 |  | 8.0 (5.0–12.0) | 5.0 (2.0–9.0) | 0.000 |
| TIL-ICPEH | Day 1 | Physician concerns of ICP/CPP | 572 | 265 | Centre distribution* | | 45 | 35 | 0.000 |
|  |  |  | 490 | 235 | Baseline functional prognosis | Pr(GOSE>4) | 36.6 (18.9–55.9) | 44.0 (21.0–63.1) | 0.008 |
|  |  |  | 490 | 235 | Baseline functional prognosis | Pr(GOSE>6) | 11.2 (5.6–19.1) | 13.7 (6.1–21.5) | 0.017 |
|  |  |  | 490 | 235 | Baseline functional prognosis | Pr(GOSE>7) | 4.4 (2.2–8.7) | 5.5 (2.4–10.2) | 0.008 |
|  |  |  | 572 | 265 | TILmax |  | 10.0 (7.0–15.0) | 10.0 (6.0–14.0) | 0.012 |
|  |  |  | 572 | 265 | TILmedian |  | 6.0 (3.0–10.0) | 5.0 (3.0–9.0) | 0.016 |
|  |  |  | 572 | 248 | TIL24 |  | 8.0 (5.0–12.0) | 6.0 (3.0–11.0) | 0.009 |
| TIL-ICPEH | Day 2 | TIL24 | 807 | 8 | Centre distribution* | | 50 | 8 | 0.000 |
|  |  |  | 707 | 7 | Baseline functional prognosis | Pr(GOSE>6) | 12.1 (6.0–19.9) | 5.0 (3.2–8.2) | 0.038 |
| TIL-ICPEH | Day 2 | ICP/CPP | 780 | 35 | Centre distribution* | | 51 | 21 | 0.003 |
|  |  |  | 688 | 26 | Baseline functional prognosis | Pr(GOSE>3) | 52.9 (29.5–74.6) | 73.4 (46.3–85.3) | 0.044 |
|  |  |  | 688 | 26 | Baseline functional prognosis | Pr(GOSE>4) | 38.6 (19.8–57.8) | 56.3 (30.8–70.9) | 0.030 |
|  |  |  | 688 | 26 | Baseline functional prognosis | Pr(GOSE>5) | 20.3 (10.0–35.3) | 40.3 (13.1–47.8) | 0.018 |
|  |  |  | 688 | 26 | Baseline functional prognosis | Pr(GOSE>6) | 11.8 (5.9–19.4) | 21.4 (7.5–32.4) | 0.019 |
|  |  |  | 688 | 26 | Baseline functional prognosis | Pr(GOSE>7) | 4.6 (2.2–8.9) | 8.3 (2.8–15.5) | 0.041 |
| TIL-ICPEH | Day 2 | Physician concerns of ICP/CPP | 603 | 212 | Centre distribution* | | 45 | 36 | 0.000 |
|  |  |  | 525 | 189 | Baseline functional prognosis | Pr(GOSE>7) | 4.6 (2.2–8.7) | 5.3 (2.3–10.5) | 0.041 |
|  |  |  | 603 | 212 | TILmax |  | 10.0 (7.0–15.0) | 9.0 (5.8–14.0) | 0.009 |
|  |  |  | 603 | 212 | TILmedian |  | 6.0 (3.0–10.0) | 5.0 (3.0–8.0) | 0.010 |
|  |  |  | 603 | 204 | TIL24 |  | 6.0 (4.0–11.0) | 6.0 (4.0–9.0) | 0.008 |
| TIL-ICPEH | Day 3 | TIL24 | 693 | 13 | Six-month GOSE | 1 | 161 (23%) | 11 (85%) | 0.000 |
|  |  |  | 693 | 13 | Six-month GOSE | 2_or_3 | 179 (26%) | 1 (8%) | 0.000 |
|  |  |  | 693 |  | Six-month GOSE | 4 | 66 (10%) |  | 0.000 |
|  |  |  | 693 | 13 | Six-month GOSE | 5 | 116 (17%) | 1 (8%) | 0.000 |
|  |  |  | 693 |  | Six-month GOSE | 6 | 67 (10%) |  | 0.000 |
|  |  |  | 693 |  | Six-month GOSE | 7 | 51 (7%) |  | 0.000 |
|  |  |  | 693 |  | Six-month GOSE | 8 | 53 (8%) |  | 0.000 |
|  |  |  | 693 | 13 | Baseline functional prognosis | Pr(GOSE>1) | 85.0 (64.5–94.9) | 40.0 (23.4–67.9) | 0.003 |
|  |  |  | 693 | 13 | Baseline functional prognosis | Pr(GOSE>3) | 54.6 (30.3–75.0) | 12.4 (7.4–39.6) | 0.003 |
|  |  |  | 693 | 13 | Baseline functional prognosis | Pr(GOSE>4) | 40.3 (21.0–58.8) | 9.5 (4.9–29.9) | 0.006 |
|  |  |  | 693 | 13 | Baseline functional prognosis | Pr(GOSE>5) | 21.2 (10.6–36.3) | 7.2 (2.2–15.5) | 0.010 |
|  |  |  | 693 | 13 | Baseline functional prognosis | Pr(GOSE>6) | 12.4 (6.1–20.0) | 3.5 (1.6–7.1) | 0.032 |
|  |  |  | 693 | 13 | Baseline functional prognosis | Pr(GOSE>7) | 4.9 (2.4–9.1) | 1.1 (0.5–4.4) | 0.001 |
|  |  |  | 788 | 13 | TILmedian |  | 5.0 (3.0–9.0) | 9.0 (7.0–14.0) | 0.007 |
| TIL-ICPEH | Day 3 | ICP/CPP | 740 | 61 | Centre distribution* | | 48 | 32 | 0.000 |
|  |  |  | 657 | 49 | Six-month GOSE | 1 | 166 (25%) | 6 (12%) | 0.034 |
|  |  |  | 657 | 49 | Six-month GOSE | 2_or_3 | 169 (26%) | 11 (22%) | 0.034 |
|  |  |  | 657 | 49 | Six-month GOSE | 4 | 64 (10%) | 2 (4%) | 0.034 |
|  |  |  | 657 | 49 | Six-month GOSE | 5 | 108 (16%) | 9 (18%) | 0.034 |
|  |  |  | 657 | 49 | Six-month GOSE | 6 | 60 (9%) | 7 (14%) | 0.034 |
|  |  |  | 657 | 49 | Six-month GOSE | 7 | 45 (7%) | 6 (12%) | 0.034 |
|  |  |  | 657 | 49 | Six-month GOSE | 8 | 45 (7%) | 8 (16%) | 0.034 |
|  |  |  | 657 | 49 | Baseline functional prognosis | Pr(GOSE>4) | 38.7 (20.8–57.1) | 58.3 (18.7–72.0) | 0.026 |
|  |  |  | 657 | 49 | Baseline functional prognosis | Pr(GOSE>5) | 20.2 (10.2–34.4) | 36.1 (10.6–48.2) | 0.010 |
|  |  |  | 657 | 49 | Baseline functional prognosis | Pr(GOSE>6) | 11.7 (6.0–18.9) | 16.1 (6.6–27.1) | 0.017 |
|  |  |  | 657 | 49 | Baseline functional prognosis | Pr(GOSE>7) | 4.6 (2.3–8.8) | 7.2 (2.7–12.9) | 0.048 |
|  |  |  | 740 | 61 | TILmax |  | 10.0 (7.0–15.0) | 7.0 (4.0–12.0) | 0.005 |
|  |  |  | 740 | 61 | TILmedian |  | 6.0 (4.0–10.0) | 3.0 (1.0–5.0) | 0.000 |
|  |  |  | 729 | 59 | TIL24 |  | 6.0 (4.0–10.0) | 3.0 (0.0–5.5) | 0.000 |
| TIL-ICPEH | Day 3 | Physician concerns of ICP/CPP | 582 | 219 | Centre distribution* | | 44 | 36 | 0.000 |
|  |  |  | 582 | 219 | TILmax |  | 10.0 (7.0–15.0) | 10.0 (6.0–14.0) | 0.020 |
|  |  |  | 582 | 219 | TILmedian |  | 6.0 (3.5–10.0) | 5.0 (3.0–8.2) | 0.037 |
|  |  |  | 582 | 206 | TIL24 |  | 6.0 (4.0–10.0) | 5.0 (3.0–8.0) | 0.000 |
| TIL-ICPEH | Day 4 | ICP/CPP | 667 | 98 | Centre distribution* | | 48 | 37 | 0.000 |
|  |  |  | 667 | 98 | Age |  | 46.0 (29.0–61.0) | 53.0 (34.2–64.8) | 0.030 |
|  |  |  | 589 | 81 | Six-month GOSE | 1 | 136 (23%) | 10 (12%) | 0.003 |
|  |  |  | 589 | 81 | Six-month GOSE | 2_or_3 | 162 (28%) | 18 (22%) | 0.003 |
|  |  |  | 589 | 81 | Six-month GOSE | 4 | 62 (11%) | 4 (5%) | 0.003 |
|  |  |  | 589 | 81 | Six-month GOSE | 5 | 100 (17%) | 16 (20%) | 0.003 |
|  |  |  | 589 | 81 | Six-month GOSE | 6 | 54 (9%) | 11 (14%) | 0.003 |
|  |  |  | 589 | 81 | Six-month GOSE | 7 | 39 (7%) | 9 (11%) | 0.003 |
|  |  |  | 589 | 81 | Six-month GOSE | 8 | 36 (6%) | 13 (16%) | 0.003 |
|  |  |  | 589 | 81 | Baseline functional prognosis | Pr(GOSE>3) | 53.4 (30.8–73.6) | 64.1 (33.6–82.3) | 0.023 |
|  |  |  | 589 | 81 | Baseline functional prognosis | Pr(GOSE>4) | 39.7 (21.3–56.5) | 49.4 (26.1–71.5) | 0.004 |
|  |  |  | 589 | 81 | Baseline functional prognosis | Pr(GOSE>5) | 20.5 (10.6–34.2) | 30.9 (14.0–48.2) | 0.001 |
|  |  |  | 589 | 81 | Baseline functional prognosis | Pr(GOSE>6) | 11.7 (6.1–18.7) | 16.5 (9.3–25.7) | 0.001 |
|  |  |  | 589 | 81 | Baseline functional prognosis | Pr(GOSE>7) | 4.6 (2.5–8.7) | 8.1 (2.7–12.6) | 0.003 |
|  |  |  | 667 | 98 | TILmax |  | 11.0 (7.0–15.0) | 6.0 (4.0–10.0) | 0.000 |
|  |  |  | 667 | 98 | TILmedian |  | 6.0 (4.0–10.0) | 2.0 (1.0–3.5) | 0.000 |
|  |  |  | 661 | 96 | TIL24 |  | 6.0 (4.0–10.0) | 1.0 (0.0–3.0) | 0.000 |
| TIL-ICPEH | Day 4 | Physician concerns of ICP/CPP | 550 | 215 | Centre distribution* | | 45 | 35 | 0.000 |
|  |  |  | 550 | 215 | TILmax |  | 10.0 (7.0–15.0) | 10.0 (6.0–14.0) | 0.009 |
|  |  |  | 550 | 215 | TILmedian |  | 6.0 (3.5–10.0) | 5.0 (3.0–8.0) | 0.001 |
|  |  |  | 550 | 207 | TIL24 |  | 6.0 (3.0–10.0) | 5.0 (3.0–8.0) | 0.003 |
| TIL-ICPEH | Day 5 | TIL24 | 642 | 10 | Six-month GOSE | 1 | 135 (21%) | 8 (80%) | 0.002 |
|  |  |  | 642 | 10 | Six-month GOSE | 2_or_3 | 175 (27%) | 2 (20%) | 0.002 |
|  |  |  | 642 |  | Six-month GOSE | 4 | 65 (10%) |  | 0.002 |
|  |  |  | 642 |  | Six-month GOSE | 5 | 114 (18%) |  | 0.002 |
|  |  |  | 642 |  | Six-month GOSE | 6 | 63 (10%) |  | 0.002 |
|  |  |  | 642 |  | Six-month GOSE | 7 | 46 (7%) |  | 0.002 |
|  |  |  | 642 |  | Six-month GOSE | 8 | 44 (7%) |  | 0.002 |
|  |  |  | 642 | 10 | Baseline functional prognosis | Pr(GOSE>1) | 85.7 (65.6–94.9) | 60.4 (35.0–69.0) | 0.016 |
|  |  |  | 642 | 10 | Baseline functional prognosis | Pr(GOSE>3) | 55.1 (31.3–75.0) | 29.0 (9.5–38.0) | 0.000 |
|  |  |  | 642 | 10 | Baseline functional prognosis | Pr(GOSE>4) | 40.7 (22.4–58.7) | 21.6 (6.0–28.6) | 0.000 |
|  |  |  | 642 | 10 | Baseline functional prognosis | Pr(GOSE>5) | 21.4 (11.0–36.3) | 10.3 (2.9–15.0) | 0.000 |
|  |  |  | 642 | 10 | Baseline functional prognosis | Pr(GOSE>6) | 12.4 (6.4–20.0) | 5.0 (2.1–8.0) | 0.001 |
|  |  |  | 642 | 10 | Baseline functional prognosis | Pr(GOSE>7) | 5.1 (2.5–9.2) | 2.3 (0.5–4.5) | 0.002 |
| TIL-ICPEH | Day 5 | ICP/CPP | 613 | 131 | Centre distribution* | | 48 | 40 | 0.002 |
|  |  |  | 503 | 103 | Marshall CT | 1 | 12 (2%) | 3 (3%) | 0.027 |
|  |  |  | 503 | 103 | Marshall CT | 2 | 177 (35%) | 53 (51%) | 0.027 |
|  |  |  | 503 | 103 | Marshall CT | 3 | 69 (14%) | 9 (9%) | 0.027 |
|  |  |  | 503 | 103 | Marshall CT | 4 | 11 (2%) | 3 (3%) | 0.027 |
|  |  |  | 503 | 103 | Marshall CT | 5_or_6 | 234 (47%) | 35 (34%) | 0.027 |
|  |  |  | 609 | 128 | Refractory intracranial hypertension | | 113 (19%) | 9 (7%) | 0.002 |
|  |  |  | 539 | 113 | Six-month GOSE | 1 | 129 (24%) | 14 (12%) | 0.036 |
|  |  |  | 539 | 113 | Six-month GOSE | 2_or_3 | 146 (27%) | 31 (27%) | 0.036 |
|  |  |  | 539 | 113 | Six-month GOSE | 4 | 58 (11%) | 7 (6%) | 0.036 |
|  |  |  | 539 | 113 | Six-month GOSE | 5 | 88 (16%) | 26 (23%) | 0.036 |
|  |  |  | 539 | 113 | Six-month GOSE | 6 | 49 (9%) | 14 (12%) | 0.036 |
|  |  |  | 539 | 113 | Six-month GOSE | 7 | 36 (7%) | 10 (9%) | 0.036 |
|  |  |  | 539 | 113 | Six-month GOSE | 8 | 33 (6%) | 11 (10%) | 0.036 |
|  |  |  | 539 | 113 | Baseline functional prognosis | Pr(GOSE>3) | 53.1 (31.1–73.0) | 61.8 (30.8–81.9) | 0.031 |
|  |  |  | 539 | 113 | Baseline functional prognosis | Pr(GOSE>4) | 39.2 (21.2–56.4) | 44.9 (24.8–69.1) | 0.011 |
|  |  |  | 539 | 113 | Baseline functional prognosis | Pr(GOSE>5) | 19.9 (10.5–33.4) | 26.8 (12.7–47.0) | 0.001 |
|  |  |  | 539 | 113 | Baseline functional prognosis | Pr(GOSE>6) | 11.6 (6.1–18.5) | 16.6 (7.8–25.5) | 0.000 |
|  |  |  | 539 | 113 | Baseline functional prognosis | Pr(GOSE>7) | 4.5 (2.3–8.5) | 7.3 (2.7–12.0) | 0.002 |
|  |  |  | 613 | 131 | TILmax |  | 11.0 (7.0–15.0) | 7.0 (4.5–10.5) | 0.000 |
|  |  |  | 613 | 131 | TILmedian |  | 6.0 (4.0–10.0) | 3.0 (1.0–4.0) | 0.000 |
|  |  |  | 603 | 128 | TIL24 |  | 6.0 (4.0–10.5) | 2.0 (1.0–3.2) | 0.000 |
| TIL-ICPEH | Day 5 | Physician concerns of ICP/CPP | 531 | 213 | Centre distribution* | | 42 | 35 | 0.000 |
|  |  |  | 531 | 213 | TILmedian |  | 6.0 (4.0–10.0) | 5.0 (3.0–8.0) | 0.009 |
|  |  |  | 531 | 200 | TIL24 |  | 6.0 (3.0–10.0) | 5.0 (2.0–9.0) | 0.001 |
| TIL-ICPEH | Day 6 | TIL24 | 612 | 9 | Six-month GOSE | 1 | 124 (20%) | 7 (78%) | 0.005 |
|  |  |  | 612 | 9 | Six-month GOSE | 2_or_3 | 174 (28%) | 2 (22%) | 0.005 |
|  |  |  | 612 |  | Six-month GOSE | 4 | 64 (10%) |  | 0.005 |
|  |  |  | 612 |  | Six-month GOSE | 5 | 107 (17%) |  | 0.005 |
|  |  |  | 612 |  | Six-month GOSE | 6 | 61 (10%) |  | 0.005 |
|  |  |  | 612 |  | Six-month GOSE | 7 | 41 (7%) |  | 0.005 |
|  |  |  | 612 |  | Six-month GOSE | 8 | 41 (7%) |  | 0.005 |
|  |  |  | 612 | 9 | Baseline functional prognosis | Pr(GOSE>1) | 85.7 (66.4–94.8) | 66.8 (38.8–75.1) | 0.046 |
|  |  |  | 612 | 9 | Baseline functional prognosis | Pr(GOSE>3) | 55.1 (32.6–74.8) | 26.9 (10.7–39.6) | 0.001 |
|  |  |  | 612 | 9 | Baseline functional prognosis | Pr(GOSE>4) | 40.8 (22.8–57.8) | 18.8 (8.5–29.8) | 0.000 |
|  |  |  | 612 | 9 | Baseline functional prognosis | Pr(GOSE>5) | 21.4 (11.2–35.6) | 11.0 (4.2–15.5) | 0.000 |
|  |  |  | 612 | 9 | Baseline functional prognosis | Pr(GOSE>6) | 12.4 (6.4–19.6) | 6.5 (2.1–8.2) | 0.005 |
|  |  |  | 612 | 9 | Baseline functional prognosis | Pr(GOSE>7) | 5.0 (2.5–8.9) | 2.6 (0.7–4.4) | 0.005 |
| TIL-ICPEH | Day 6 | ICP/CPP | 547 | 167 | Centre distribution* | | 48 | 39 | 0.000 |
|  |  |  | 547 | 167 | Age |  | 44.0 (28.0–59.5) | 51.0 (34.0–65.5) | 0.004 |
|  |  |  | 543 | 164 | Refractory intracranial hypertension | | 110 (20%) | 10 (6%) | 0.000 |
|  |  |  | 480 | 141 | Six-month GOSE | 1 | 106 (22%) | 25 (18%) | 0.028 |
|  |  |  | 480 | 141 | Six-month GOSE | 2_or_3 | 137 (29%) | 39 (28%) | 0.028 |
|  |  |  | 480 | 141 | Six-month GOSE | 4 | 57 (12%) | 7 (5%) | 0.028 |
|  |  |  | 480 | 141 | Six-month GOSE | 5 | 73 (15%) | 34 (24%) | 0.028 |
|  |  |  | 480 | 141 | Six-month GOSE | 6 | 45 (9%) | 16 (11%) | 0.028 |
|  |  |  | 480 | 141 | Six-month GOSE | 7 | 34 (7%) | 7 (5%) | 0.028 |
|  |  |  | 480 | 141 | Six-month GOSE | 8 | 28 (6%) | 13 (9%) | 0.028 |
|  |  |  | 480 | 141 | Baseline functional prognosis | Pr(GOSE>6) | 12.2 (6.1–18.6) | 12.9 (7.3–21.6) | 0.044 |
|  |  |  | 547 | 167 | TILmax |  | 11.0 (8.0–16.0) | 7.0 (5.0–11.0) | 0.000 |
|  |  |  | 547 | 167 | TILmedian |  | 7.0 (4.0–10.0) | 3.0 (2.0–5.0) | 0.000 |
|  |  |  | 537 | 166 | TIL24 |  | 6.0 (4.0–10.0) | 2.0 (1.0–3.0) | 0.000 |
| TIL-ICPEH | Day 6 | Physician concerns of ICP/CPP | 509 | 205 | Centre distribution* | | 42 | 34 | 0.000 |
|  |  |  | 509 | 205 | TILmax |  | 11.0 (7.0–15.0) | 10.0 (6.0–14.0) | 0.021 |
|  |  |  | 509 | 205 | TILmedian |  | 6.0 (4.0–10.0) | 5.0 (3.0–8.0) | 0.002 |
|  |  |  | 509 | 194 | TIL24 |  | 5.0 (3.0–10.0) | 5.0 (2.0–8.0) | 0.006 |
| TIL-ICPEH | Day 7 | TIL24 | 679 | 13 | Centre distribution* | | 50 | 9 | 0.008 |
| TIL-ICPEH | Day 7 | ICP/CPP | 433 | 259 | Centre distribution* | | 44 | 43 | 0.000 |
|  |  |  | 359 | 208 | Marshall CT | 1 | 7 (2%) | 7 (3%) | 0.015 |
|  |  |  | 359 | 208 | Marshall CT | 2 | 119 (33%) | 96 (46%) | 0.015 |
|  |  |  | 359 | 208 | Marshall CT | 3 | 53 (15%) | 19 (9%) | 0.015 |
|  |  |  | 359 | 208 | Marshall CT | 4 | 9 (3%) | 4 (2%) | 0.015 |
|  |  |  | 359 | 208 | Marshall CT | 5_or_6 | 171 (48%) | 82 (39%) | 0.015 |
|  |  |  | 430 | 255 | Refractory intracranial hypertension | | 98 (23%) | 17 (7%) | 0.000 |
|  |  |  | 380 | 223 | Six-month GOSE | 1 | 89 (23%) | 33 (15%) | 0.001 |
|  |  |  | 380 | 223 | Six-month GOSE | 2_or_3 | 112 (29%) | 62 (28%) | 0.001 |
|  |  |  | 380 | 223 | Six-month GOSE | 4 | 44 (12%) | 20 (9%) | 0.001 |
|  |  |  | 380 | 223 | Six-month GOSE | 5 | 49 (13%) | 56 (25%) | 0.001 |
|  |  |  | 380 | 223 | Six-month GOSE | 6 | 37 (10%) | 22 (10%) | 0.001 |
|  |  |  | 380 | 223 | Six-month GOSE | 7 | 30 (8%) | 11 (5%) | 0.001 |
|  |  |  | 380 | 223 | Six-month GOSE | 8 | 19 (5%) | 19 (9%) | 0.001 |
|  |  |  | 380 | 223 | Baseline functional prognosis | Pr(GOSE>1) | 82.5 (63.8–93.4) | 90.1 (73.2–96.4) | 0.000 |
|  |  |  | 380 | 223 | Baseline functional prognosis | Pr(GOSE>3) | 51.4 (29.3–72.4) | 60.0 (38.9–78.4) | 0.000 |
|  |  |  | 380 | 223 | Baseline functional prognosis | Pr(GOSE>4) | 37.3 (19.3–55.1) | 43.9 (28.1–63.5) | 0.000 |
|  |  |  | 380 | 223 | Baseline functional prognosis | Pr(GOSE>5) | 19.6 (9.8–33.2) | 23.7 (13.7–38.4) | 0.004 |
|  |  |  | 380 | 223 | Baseline functional prognosis | Pr(GOSE>6) | 11.4 (5.6–18.3) | 13.3 (7.9–21.6) | 0.004 |
|  |  |  | 380 | 223 | Baseline functional prognosis | Pr(GOSE>7) | 4.4 (2.2–8.2) | 5.8 (3.2–10.4) | 0.005 |
|  |  |  | 433 | 259 | TILmax |  | 12.0 (9.0–16.0) | 8.0 (5.0–12.0) | 0.000 |
|  |  |  | 433 | 259 | TILmedian |  | 7.0 (5.0–11.0) | 4.0 (2.0–6.0) | 0.000 |
|  |  |  | 426 | 253 | TIL24 |  | 6.0 (4.0–11.0) | 2.0 (1.0–4.0) | 0.000 |
| TIL-ICPEH | Day 7 | Physician concerns of ICP/CPP | 483 | 209 | Centre distribution* | | 41 | 38 | 0.000 |
|  |  |  | 483 | 209 | TILmedian |  | 6.0 (4.0–10.0) | 5.0 (3.0–8.0) | 0.013 |
|  |  |  | 483 | 196 | TIL24 |  | 5.0 (2.0–9.0) | 4.0 (2.0–7.0) | 0.022 |
| TIL-ICPHR | Day 1 | TIL24 | 256 | 3 | Centre distribution* | | 21 | 2 | 0.006 |
|  |  |  | 256 | 3 | TILmax |  | 10.0 (6.0–14.0) | 6.0 (5.5–7.0) | 0.023 |
| TIL-ICPHR | Day 1 | ICP/CPP | 21 | 238 | Centre distribution* | | 5 | 21 | 0.000 |
|  |  |  | 21 | 238 | Age |  | 64.0 (51.0–70.0) | 46.5 (30.0–61.0) | 0.008 |
|  |  |  | 20 | 213 | Baseline functional prognosis | Pr(GOSE>3) | 34.4 (24.8–49.7) | 54.2 (35.8–72.5) | 0.009 |
|  |  |  | 20 | 213 | Baseline functional prognosis | Pr(GOSE>4) | 25.5 (14.1–37.5) | 39.8 (23.2–55.1) | 0.008 |
|  |  |  | 20 | 213 | Baseline functional prognosis | Pr(GOSE>5) | 13.3 (6.9–22.2) | 19.6 (10.6–31.5) | 0.011 |
| TIL-ICPHR | Day 1 | Physician concerns of ICP/CPP | 192 | 67 | Centre distribution* | | 17 | 13 | 0.000 |
|  |  |  | 174 | 59 | Baseline functional prognosis | Pr(GOSE>6) | 10.1 (5.2–16.4) | 13.7 (8.7–21.5) | 0.007 |
|  |  |  | 174 | 59 | Baseline functional prognosis | Pr(GOSE>7) | 4.4 (2.0–7.8) | 7.4 (3.3–12.0) | 0.001 |
| TIL-ICPHR | Day 2 | ICP/CPP | 168 | 89 | Centre distribution* | | 19 | 19 | 0.000 |
|  |  |  | 168 | 89 | Age |  | 51.0 (32.0–64.0) | 43.0 (27.0–60.0) | 0.018 |
|  |  |  | 167 | 89 | TIL24 |  | 6.0 (4.0–9.0) | 6.0 (5.0–11.0) | 0.048 |
| TIL-ICPHR | Day 2 | Physician concerns of ICP/CPP | 209 | 48 | Centre distribution* | | 16 | 16 | 0.000 |
|  |  |  | 191 | 40 | Baseline functional prognosis | Pr(GOSE>6) | 10.5 (5.6–16.7) | 13.5 (7.3–22.2) | 0.045 |
|  |  |  | 191 | 40 | Baseline functional prognosis | Pr(GOSE>7) | 4.8 (2.2–8.2) | 6.4 (3.1–12.7) | 0.043 |
|  |  |  | 209 | 48 | TILmax |  | 10.0 (6.0–15.0) | 9.0 (5.8–11.0) | 0.017 |
|  |  |  | 209 | 47 | TIL24 |  | 6.0 (5.0–10.0) | 5.0 (3.0–8.5) | 0.003 |
| TIL-ICPHR | Day 3 | TIL24 | 226 | 5 | Six-month GOSE | 1 | 47 (21%) | 5 (100%) | 0.007 |
|  |  |  | 226 |  | Six-month GOSE | 2_or_3 | 63 (28%) |  | 0.007 |
|  |  |  | 226 |  | Six-month GOSE | 4 | 22 (10%) |  | 0.007 |
|  |  |  | 226 |  | Six-month GOSE | 5 | 44 (19%) |  | 0.007 |
|  |  |  | 226 |  | Six-month GOSE | 6 | 23 (10%) |  | 0.007 |
|  |  |  | 226 |  | Six-month GOSE | 7 | 14 (6%) |  | 0.007 |
|  |  |  | 226 |  | Six-month GOSE | 8 | 13 (6%) |  | 0.007 |
|  |  |  | 226 | 5 | Baseline functional prognosis | Pr(GOSE>3) | 53.7 (35.2–72.2) | 33.2 (12.4–39.6) | 0.040 |
|  |  |  | 226 | 5 | Baseline functional prognosis | Pr(GOSE>4) | 38.9 (23.3–55.0) | 25.2 (7.9–29.9) | 0.022 |
|  |  |  | 226 | 5 | Baseline functional prognosis | Pr(GOSE>5) | 19.6 (10.7–31.0) | 12.9 (4.6–18.9) | 0.029 |
|  |  |  | 226 | 5 | Baseline functional prognosis | Pr(GOSE>6) | 11.7 (6.0–17.5) | 7.1 (3.5–8.3) | 0.017 |
|  |  |  | 226 | 5 | Baseline functional prognosis | Pr(GOSE>7) | 5.5 (2.3–8.6) | 3.8 (1.2–4.4) | 0.009 |
|  |  |  | 251 | 5 | TILmedian |  | 5.0 (3.2–10.0) | 9.0 (8.5–9.5) | 0.018 |
| TIL-ICPHR | Day 3 | ICP/CPP | 242 | 14 | Sex | F | 47 (19%) | 7 (50%) | 0.017 |
|  |  |  | 237 | 14 | TIL24 |  | 6.0 (4.0–9.0) | 3.0 (2.2–6.5) | 0.024 |
| TIL-ICPHR | Day 3 | Physician concerns of ICP/CPP | 203 | 53 | Centre distribution* | | 17 | 16 | 0.000 |
|  |  |  | 185 | 46 | Baseline functional prognosis | Pr(GOSE>6) | 10.3 (5.5–16.5) | 13.6 (7.9–23.6) | 0.017 |
|  |  |  | 185 | 46 | Baseline functional prognosis | Pr(GOSE>7) | 4.6 (2.2–8.1) | 6.8 (3.7–13.0) | 0.015 |
| TIL-ICPHR | Day 4 | TIL24 | 219 | 5 | Baseline functional prognosis | Pr(GOSE>3) | 53.9 (35.7–72.4) | 26.9 (12.4–33.2) | 0.008 |
|  |  |  | 219 | 5 | Baseline functional prognosis | Pr(GOSE>4) | 39.2 (24.4–55.0) | 18.5 (7.9–25.2) | 0.008 |
|  |  |  | 219 | 5 | Baseline functional prognosis | Pr(GOSE>5) | 20.2 (11.7–31.2) | 7.6 (4.6–12.9) | 0.007 |
|  |  |  | 219 | 5 | Baseline functional prognosis | Pr(GOSE>6) | 11.9 (6.1–17.6) | 3.5 (3.5–8.3) | 0.012 |
|  |  |  | 219 | 5 | Baseline functional prognosis | Pr(GOSE>7) | 5.6 (2.5–8.7) | 1.2 (0.8–4.4) | 0.006 |
| TIL-ICPHR | Day 4 | ICP/CPP | 221 | 28 | Centre distribution* | | 21 | 13 | 0.016 |
|  |  |  | 221 | 28 | Sex | F | 41 (19%) | 11 (39%) | 0.022 |
|  |  |  | 199 | 25 | Six-month GOSE | 1 | 41 (21%) | 5 (20%) | 0.043 |
|  |  |  | 199 | 25 | Six-month GOSE | 2_or_3 | 57 (29%) | 6 (24%) | 0.043 |
|  |  |  | 199 |  | Six-month GOSE | 4 | 22 (11%) |  | 0.043 |
|  |  |  | 199 | 25 | Six-month GOSE | 5 | 38 (19%) | 5 (20%) | 0.043 |
|  |  |  | 199 | 25 | Six-month GOSE | 6 | 21 (11%) | 2 (8%) | 0.043 |
|  |  |  | 199 | 25 | Six-month GOSE | 7 | 12 (6%) | 2 (8%) | 0.043 |
|  |  |  | 199 | 25 | Six-month GOSE | 8 | 8 (4%) | 5 (20%) | 0.043 |
|  |  |  | 221 | 28 | TILmax |  | 10.0 (7.0–14.0) | 5.0 (4.0–8.2) | 0.009 |
|  |  |  | 221 | 28 | TILmedian |  | 6.0 (4.0–10.0) | 2.0 (1.0–4.2) | 0.001 |
|  |  |  | 217 | 27 | TIL24 |  | 6.0 (4.0–10.0) | 2.0 (0.5–5.0) | 0.020 |
| TIL-ICPHR | Day 4 | Physician concerns of ICP/CPP | 195 | 54 | Centre distribution* | | 17 | 14 | 0.000 |
| TIL-ICPHR | Day 5 | TIL24 | 215 | 6 | Six-month GOSE | 1 | 41 (19%) | 5 (83%) | 0.020 |
|  |  |  | 215 | 6 | Six-month GOSE | 2_or_3 | 61 (28%) | 1 (17%) | 0.020 |
|  |  |  | 215 |  | Six-month GOSE | 4 | 22 (10%) |  | 0.020 |
|  |  |  | 215 |  | Six-month GOSE | 5 | 43 (20%) |  | 0.020 |
|  |  |  | 215 |  | Six-month GOSE | 6 | 23 (11%) |  | 0.020 |
|  |  |  | 215 |  | Six-month GOSE | 7 | 14 (7%) |  | 0.020 |
|  |  |  | 215 |  | Six-month GOSE | 8 | 11 (5%) |  | 0.020 |
|  |  |  | 215 | 6 | Baseline functional prognosis | Pr(GOSE>3) | 53.9 (35.7–72.4) | 19.7 (9.5–31.6) | 0.003 |
|  |  |  | 215 | 6 | Baseline functional prognosis | Pr(GOSE>4) | 39.2 (24.8–55.0) | 13.2 (5.6–23.6) | 0.002 |
|  |  |  | 215 | 6 | Baseline functional prognosis | Pr(GOSE>5) | 20.2 (11.7–31.2) | 6.1 (2.8–11.6) | 0.002 |
|  |  |  | 215 | 6 | Baseline functional prognosis | Pr(GOSE>6) | 11.9 (6.1–17.6) | 3.5 (2.1–7.1) | 0.003 |
|  |  |  | 215 | 6 | Baseline functional prognosis | Pr(GOSE>7) | 5.6 (2.5–8.7) | 1.0 (0.5–3.6) | 0.001 |
| TIL-ICPHR | Day 5 | ICP/CPP | 193 | 53 | Centre distribution* | | 20 | 17 | 0.001 |
|  |  |  | 193 | 53 | TILmax |  | 11.0 (7.0–15.0) | 7.0 (5.0–9.0) | 0.000 |
|  |  |  | 193 | 53 | TILmedian |  | 6.0 (4.0–10.0) | 3.0 (2.0–5.0) | 0.000 |
|  |  |  | 188 | 51 | TIL24 |  | 6.0 (3.8–11.0) | 3.0 (1.0–4.0) | 0.000 |
| TIL-ICPHR | Day 5 | Physician concerns of ICP/CPP | 191 | 55 | Centre distribution* | | 17 | 14 | 0.000 |
| TIL-ICPHR | Day 6 | TIL24 | 204 | 4 | Baseline functional prognosis | Pr(GOSE>3) | 53.3 (35.7–70.8) | 17.8 (6.7–30.1) | 0.032 |
|  |  |  | 204 | 4 | Baseline functional prognosis | Pr(GOSE>4) | 38.9 (24.9–54.6) | 11.7 (3.9–21.4) | 0.028 |
|  |  |  | 204 | 4 | Baseline functional prognosis | Pr(GOSE>5) | 19.6 (11.8–29.9) | 4.9 (1.8–10.6) | 0.033 |
|  |  |  | 204 | 4 | Baseline functional prognosis | Pr(GOSE>6) | 11.5 (6.1–16.8) | 2.6 (1.2–5.7) | 0.042 |
|  |  |  | 204 | 4 | Baseline functional prognosis | Pr(GOSE>7) | 5.4 (2.5–8.4) | 0.6 (0.4–1.7) | 0.012 |
| TIL-ICPHR | Day 6 | ICP/CPP | 170 | 63 | Centre distribution* | | 20 | 15 | 0.000 |
|  |  |  | 170 | 63 | Refractory intracranial hypertension | | 35 (21%) | 5 (8%) | 0.038 |
|  |  |  | 170 | 63 | TILmax |  | 11.0 (8.0–16.0) | 7.0 (5.0–10.0) | 0.000 |
|  |  |  | 170 | 63 | TILmedian |  | 6.5 (4.0–10.0) | 4.0 (3.0–6.0) | 0.000 |
|  |  |  | 168 | 60 | TIL24 |  | 6.0 (4.0–10.0) | 3.0 (1.0–6.0) | 0.000 |
| TIL-ICPHR | Day 6 | Physician concerns of ICP/CPP | 183 | 50 | Centre distribution* | | 17 | 16 | 0.000 |
| TIL-ICPHR | Day 7 | ICP/CPP | 130 | 96 | Centre distribution* | | 20 | 18 | 0.013 |
|  |  |  | 130 | 96 | Refractory intracranial hypertension | | 28 (22%) | 10 (10%) | 0.042 |
|  |  |  | 117 | 85 | Baseline functional prognosis | Pr(GOSE>1) | 82.5 (63.8–94.0) | 85.9 (70.3–94.0) | 0.042 |
|  |  |  | 130 | 96 | TILmax |  | 12.0 (8.0–16.0) | 8.0 (5.0–11.0) | 0.000 |
|  |  |  | 130 | 96 | TILmedian |  | 7.0 (4.1–11.0) | 4.5 (3.0–8.0) | 0.000 |
|  |  |  | 128 | 93 | TIL24 |  | 6.0 (4.0–10.0) | 3.0 (1.0–6.0) | 0.000 |
| TIL-ICPHR | Day 7 | Physician concerns of ICP/CPP | 171 | 55 | Centre distribution* | | 17 | 16 | 0.000 |

Abbreviations: Baseline GCS=Glasgow Coma Scale at ICU admission, from 3 to 15, GOS=Glasgow Outcome Scale, ICP=intracranial pressure, ICP_EH_=end-hour ICP, ICP_HR_=high-resolution ICP, Marshall CT=Marshall computerised tomography classification, Pr(GOSE>•)=“probability of GOSE greater than • at six months post-injury” as previously calculated from the first 24 hours of admission,^27^ TIL=Therapy Intensity Level scale, TIL_24_=TIL score of calendar day in ICU, TIL_max_=maximum TIL_24_ over first week of ICU stay, TIL_median_=median TIL_24_ over first week of ICU stay. Data are median (IQR) for numeric characteristics and *n* (% of column group) for categorical characteristics, unless otherwise indicated.

*The values for centre distribution represent the number of unique centres for the non-missing value and missing value cohorts, respectively. For statistical testing, centre affiliation was treated as a categorical variable with 52 possible values.

^†^*p*-values, comparing patients in non-missing value group to those in the missing value group, are derived from with Welch’s *t*-test for numeric variables and χ^2^ contingency table test for categorical variables
