# Supplementary material for: Therapy Intensity Level Scale for Traumatic Brain Injury: Clinimetric Assessment on Neuro-Monitored Patients Across 52 European Intensive Care Units
Source: J Neurotrauma. 2024 Apr 4;41(7-8):887–909. doi: 10.1089/neu.2023.0377 (PMC11005383; doi:10.1089/neu.2023.0377)
Supplement: Supplemental data [file Suppl_TableS2.docx]

**Supplementary Table S2. Study population representation of each TIL item and sub-item.**

| **ICP-treatment modality** | **Item** | | **TIL validation population** | | |
| --- | --- | --- | --- | --- | --- |
|  |  | **Sub-item** | **Overall** (*n*=873, 52 centres) | **TIL-ICP_EH_** (*n*=832, 51 centres) | **TIL-ICP_HR_** (*n*=255, 21 centres) |
| Positioning | Head elevation for ICP control or nursed flat (180°) for CPP management* | | 827 (94.7%) | 793 (95.3%) | 245 (96.1%) |
| Sedation and neuromuscular blockade | Sedation* | | 851 (97.5%) | 819 (98.4%) | 253 (99.2%) |
|  |  | Low dose sedation (as required for mechanical ventilation) | 592 (67.8%) | 579 (69.6%) | 198 (77.6%) |
|  |  | Higher dose sedation for ICP control (but not aiming for burst suppression) | 479 (54.9%) | 464 (55.8%) | 148 (58.0%) |
|  |  | High dose propofol or barbiturates for ICP control (metabolic suppression) | 309 (35.4%) | 291 (35.0%) | 64 (25.1%) |
|  | Neuromuscular blockade (paralysis)* | | 305 (34.9%) | 291 (35.0%) | 78 (30.6%) |
| CSF drainage | CSF drainage volume* | | 273 (31.3%) | 256 (30.8%) | 66 (25.9%) |
|  |  | Low (<120 ml/day) | 208 (23.8%) | 197 (23.7%) | 50 (19.6%) |
|  |  | High (≥120 ml/day) | 186 (21.3%) | 174 (20.9%) | 44 (17.3%) |
| CPP management | Fluid loading for maintenance of cerebral perfusion* | | 517 (59.2%) | 503 (60.5%) | 172 (67.5%) |
|  | Vasopressor therapy required for management of cerebral perfusion* | | 738 (84.5%) | 716 (86.1%) | 220 (86.3%) |
| Ventilatory management | Hypocapnia for ICP control (P_a_CO_2_ in mmHg)* | | 561 (64.3%) | 540 (64.9%) | 191 (74.9%) |
|  |  | Mild (35≤P_a_CO_2_<40) | 496 (56.8%) | 478 (57.5%) | 177 (69.4%) |
|  |  | Moderate (30≤P_a_CO_2_<35) | 214 (24.5%) | 208 (25.0%) | 74 (29.0%) |
|  |  | Intensive (P_a_CO_2_<30) | 57 (6.5%) | 57 (6.9%) | 17 (6.7%) |
| Hyperosmolar therapy | Mannitol* | | 238 (27.3%) | 232 (27.9%) | 69 (27.1%) |
|  |  | ≤2g/kg/24h | 224 (25.7%) | 218 (26.2%) | 67 (26.3%) |
|  |  | >2g/kg/24h | 43 (4.9%) | 42 (5.0%) | 5 (2.0%) |
|  | Hypertonic saline* | | 350 (40.1%) | 341 (41.0%) | 113 (44.3%) |
|  |  | ≤0.3g/kg/24h | 312 (35.7%) | 306 (36.8%) | 105 (41.2%) |
|  |  | >0.3g/kg/24h | 115 (13.2%) | 111 (13.3%) | 35 (13.7%) |
| Temperature control | Temperature control* | | 575 (65.9%) | 555 (66.7%) | 190 (74.5%) |
|  |  | Fever control (>38 or spontaneous <34.5) | 484 (55.4%) | 465 (55.9%) | 163 (63.9%) |
|  |  | Cooling for ICP control (≥35) | 128 (14.7%) | 125 (15.0%) | 50 (19.6%) |
|  |  | Hypothermia (<35) | 94 (10.8%) | 93 (11.2%) | 22 (8.6%) |
| Surgery for intracranial hypertension | Intracranial operation for progressive mass lesion, NOT scheduled on admission* | | 147 (16.8%) | 144 (17.3%) | 52 (20.4%) |
|  | Decompressive craniectomy* | | 196 (22.5%) | 189 (22.7%) | 56 (22.0%) |

Abbreviations: CPP=cerebral perfusion pressure, CSF=cerebrospinal fluid, ICP=intracranial pressure, ICP_EH_=end-hour ICP, ICP_HR_=high-resolution ICP, P_a_CO_2_=partial pressure of carbon dioxide in arterial blood, PILOT=Paediatric Intensity Level of Therapy scale,^7^ T=body temperature in degrees Celsius, TIL=Therapy Intensity Level scale,^8,9^ TIL^(1987)^=original Therapy Intensity Level scale published in 1987,^6^ TIL^(Basic)^=condensed TIL scale,^8^ uwTIL=unweighted TIL scale in which sub-item scores are replaced by the ascending rank index within the item.

Data are number of unique patients (% of column population) who received the item or sub-item therapy during the first week of their ICU stays.

*Data in these rows represent the number of unique patients (% of column population) who received any of the sub-item therapies for a given item during the first week of their ICU stays.
