# Supplementary material for: Therapy Intensity Level Scale for Traumatic Brain Injury: Clinimetric Assessment on Neuro-Monitored Patients Across 52 European Intensive Care Units
Source: J Neurotrauma. 2024 Apr 4;41(7-8):887–909. doi: 10.1089/neu.2023.0377 (PMC11005383; doi:10.1089/neu.2023.0377)
Supplement: Supplemental data [file Suppl_FigS3.pdf]

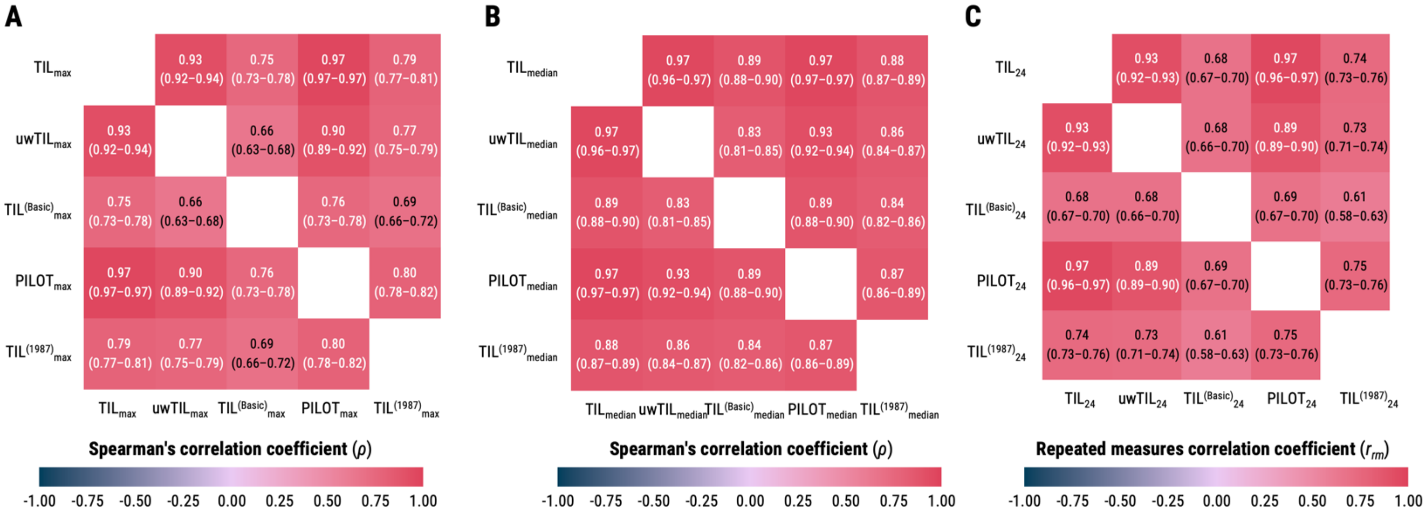

Supplementary Figure S3. Correlation matrices between total scores of TIL and alternative scales. Abbreviations: ICU=intensive care unit, PILOT=Paediatric Intensity Level of Therapy scale,[7] TIL=Therapy Intensity Level scale,[8,9] $TIL^{(1987)}$=original Therapy Intensity Level scale published in 1987,[6] $TIL^{(Basic)}$=condensed TIL scale,[8] uwTIL=unweighted TIL scale in which sub-item scores are replaced by the ascending rank index within the item. The numeric definition of each scale is listed in Table 1, and the calculation of daily (e.g., TIL_{24}), maximum (e.g., TIL_{max}), and median (e.g., TIL_{median}) scores are described in the Methods. The values in parentheses represent 95% confidence intervals derived from bootstrapping with 1,000 resamples of unique patients over 100 missing value imputations. (**A**) Spearman's correlation matrix between maximum total scores of TIL and alternative scales. (**B**) Spearman's correlation matrix between median total scores of TIL and alternative scales. (**C**) Repeated measures (i.e., within-individual) correlation matrix between daily total scores of TIL and alternative scales.
