# Supplementary material for: Therapy Intensity Level Scale for Traumatic Brain Injury: Clinimetric Assessment on Neuro-Monitored Patients Across 52 European Intensive Care Units
Source: J Neurotrauma. 2024 Apr 4;41(7-8):887–909. doi: 10.1089/neu.2023.0377 (PMC11005383; doi:10.1089/neu.2023.0377)
Supplement: Supplemental data [file Suppl_TableS3.docx]

**Supplementary Table S3. Performance of refractory intracranial hypertension status detection at each threshold of TIL_max_, TIL_median_, TIL^(Basic)^_max_, and TIL^(Basic)^_median_.**

| **Scale summary metric** | **Threshold** (≥) | **Sensitivity** (%) | **Specificity** (%) | **Youden’s J** (%) |
| --- | --- | --- | --- | --- |
| TIL_max_ | 0 | 100 (100–100) | 0 (0–0) | 0 (0–0) |
|  | 1 | 100 (99–100) | 0 (0–1) | 0 (-1–1) |
|  | 2 | 99 (98–100) | 1 (0–1) | -0 (-2–1) |
|  | 3 | 99 (98–100) | 3 (2–4) | 2 (0–4) |
|  | 4 | 99 (98–100) | 8 (6–9) | 7 (4–9) |
|  | 5 | 98 (97–100) | 15 (13–17) | 13 (10–16) |
|  | 6 | 96 (94–98) | 23 (20–25) | 19 (15–22) |
|  | 7 | 94 (92–98) | 31 (28–33) | 25 (21–29) |
|  | 8 | 94 (91–97) | 39 (36–42) | 33 (29–37) |
|  | 9 | 91 (88–95) | 45 (42–48) | 36 (32–40) |
|  | 10 | 88 (84–92) | 53 (50–56) | 41 (36–46) |
|  | 11 | 83 (78–87) | 61 (58–64) | 43 (38–49) |
|  | 12 | 77 (72–82) | 67 (64–70) | 43 (38–49) |
|  | 13 | 73 (68–78) | 73 (71–76) | 46 (41–53) |
|  | **14** | **68 (62–74)** | **79 (77–81)** | **47 (41–53)** |
|  | 15 | 64 (58–70) | 83 (81–85) | 47 (40–53) |
|  | 16 | 56 (50–62) | 86 (84–88) | 42 (36–48) |
|  | 17 | 50 (44–56) | 90 (88–91) | 39 (33–46) |
|  | 18 | 44 (38–50) | 93 (91–94) | 37 (31–43) |
|  | 19 | 38 (32–44) | 94 (93–96) | 32 (26–38) |
|  | 20 | 32 (27–38) | 96 (95–97) | 29 (23–34) |
|  | 21 | 27 (22–33) | 97 (96–98) | 24 (19–30) |
|  | 22 | 24 (19–29) | 98 (97–99) | 22 (17–27) |
|  | 23 | 21 (16–26) | 98 (98–99) | 19 (14–24) |
|  | 24 | 14 (10–18) | 99 (98–99) | 13 (9–17) |
|  | 25 | 10 (6–14) | 99 (99–100) | 9 (6–13) |
|  | 26 | 7 (4–10) | 100 (99–100) | 6 (3–9) |
|  | 27 | 5 (2–7) | 100 (100–100) | 4 (2–7) |
|  | 28 | 3 (1–4) | 100 (100–100) | 3 (1–4) |
|  | 29 | 2 (0–3) | 100 (100–100) | 2 (0–3) |
|  | 30 | 1 (0–2) | 100 (100–100) | 1 (0–2) |
|  | 31 | 1 (0–1) | 100 (100–100) | 1 (0–1) |
| TIL_median_ | 0 | 100 (100–100) | 0 (0–0) | 0 (0–0) |
|  | 0.5 | 99 (98–100) | 4 (3–5) | 4 (2–5) |
|  | 1 | 99 (98–100) | 5 (3–6) | 4 (2–5) |
|  | 1.5 | 98 (97–100) | 10 (8–11) | 8 (5–10) |
|  | 2 | 98 (97–100) | 10 (8–12) | 8 (6–11) |
|  | 2.5 | 97 (95–99) | 18 (16–20) | 15 (12–18) |
|  | 3 | 97 (95–99) | 18 (16–21) | 15 (12–18) |
|  | 3.5 | 95 (93–98) | 32 (29–34) | 27 (24–31) |
|  | 4 | 95 (93–98) | 32 (30–35) | 28 (24–32) |
|  | 4.5 | 92 (89–96) | 45 (42–48) | 37 (33–42) |
|  | 5 | 92 (89–95) | 45 (42–48) | 37 (33–42) |
|  | 5.5 | 88 (84–92) | 59 (56–62) | 47 (42–52) |
|  | 6 | 88 (84–92) | 59 (56–62) | 47 (43–52) |
|  | 6.5 | 84 (81–88) | 68 (66–69) | 52 (49–56) |
|  | 7 | 84 (80–89) | 67 (65–70) | 52 (46–57) |
|  | **7.5** | **81 (77–87)** | **72 (70–75)** | **54 (48–60)** |
|  | 8 | 81 (76–86) | 72 (70–75) | 53 (47–59) |
|  | 8.5 | 73 (68–79) | 79 (77–80) | 52 (47–58) |
|  | 9 | 73 (68–79) | 78 (76–81) | 52 (46–58) |
|  | 9.5 | 65 (58–72) | 82 (81–84) | 48 (41–55) |
|  | 10 | 65 (59–71) | 82 (80–85) | 47 (41–54) |
|  | 10.5 | 59 (53–65) | 87 (85–89) | 46 (40–53) |
|  | 11 | 59 (53–65) | 87 (86–89) | 47 (40–53) |
|  | 11.5 | 52 (46–57) | 91 (90–93) | 43 (36–48) |
|  | 12 | 52 (46–58) | 91 (90–93) | 43 (36–49) |
|  | 12.5 | 44 (38–50) | 94 (93–96) | 38 (31–44) |
|  | 13 | 43 (37–49) | 94 (93–96) | 37 (31–43) |
|  | 13.5 | 36 (30–41) | 96 (95–97) | 32 (25–37) |
|  | 14 | 35 (29–40) | 96 (95–97) | 31 (25–36) |
|  | 14.5 | 31 (26–37) | 97 (96–98) | 28 (23–34) |
|  | 15 | 31 (25–36) | 97 (96–98) | 28 (22–34) |
|  | 15.5 | 24 (19–29) | 98 (98–99) | 22 (17–27) |
|  | 16 | 23 (18–28) | 98 (98–99) | 22 (16–27) |
|  | 16.5 | 20 (15–23) | 99 (99–100) | 19 (15–23) |
|  | 17 | 19 (14–24) | 99 (99–100) | 18 (14–23) |
|  | 17.5 | 13 (9–18) | 100 (99–100) | 13 (9–17) |
|  | 18 | 13 (9–18) | 100 (100–100) | 13 (9–17) |
|  | 18.5 | 9 (7–11) | 100 (100–100) | 9 (7–11) |
|  | 19 | 8 (4–11) | 100 (100–100) | 7 (4–11) |
|  | 19.5 | 7 (6–9) | 100 (100–100) | 7 (6–9) |
|  | 20 | 6 (3–8) | 100 (100–100) | 6 (3–8) |
|  | 20.5 | 6 (2–8) | 100 (100–100) | 5 (2–8) |
|  | 21 | 5 (1–7) | 100 (100–100) | 5 (1–7) |
|  | 21.5 | 6 (6–6) | 100 (100–100) | 6 (6–6) |
|  | 22 | 5 (2–7) | 100 (100–100) | 5 (2–7) |
|  | 22.5 | 3 (1–5) | 100 (100–100) | 3 (1–4) |
|  | 23 | 3 (1–5) | 100 (100–100) | 3 (1–5) |
|  | 24 | 1 (0–2) | 100 (100–100) | 1 (0–2) |
|  | 25 | 0 (0–0) | 100 (100–100) | 0 (0–0) |
|  | 27 | 1 (1–1) | 100 (100–100) | 1 (1–1) |
|  | 28 | 0 (0–0) | 100 (100–100) | 0 (0–0) |
| TIL^(Basic)^_max_ | 0 | 100 (100–100) | 0 (0–0) | 0 (0–0) |
|  | 1 | 100 (99–100) | 0 (0–1) | 0 (-1–1) |
|  | 2 | 99 (98–100) | 3 (2–4) | 2 (0–3) |
|  | 3 | 94 (91–97) | 32 (29–35) | 26 (22–30) |
|  | **4** | **85 (81–89)** | **49 (45–52)** | **33 (28–39)** |
|  | 5 | 0 (0–0) | 100 (100–100) | 0 (0–0) |
| TIL^(Basic)^_median_ | 0 | 100 (100–100) | 0 (0–0) | 0 (0–0) |
|  | 0.5 | 99 (98–100) | 5 (4–6) | 5 (3–6) |
|  | 1 | 99 (98–100) | 6 (4–7) | 5 (3–7) |
|  | 1.5 | 98 (97–100) | 16 (14–18) | 15 (12–17) |
|  | 2 | 98 (97–100) | 18 (15–20) | 16 (13–18) |
|  | 2.5 | 80 (75–85) | 67 (64–69) | 47 (41–52) |
|  | 3 | 79 (74–84) | 67 (65–70) | 46 (41–52) |
|  | **3.5** | **67 (61–72)** | **81 (79–84)** | **48 (42–54)** |
|  | 4 | 65 (60–71) | 81 (79–84) | 47 (41–52) |
|  | 5 | 0 (0–0) | 100 (100–100) | 0 (0–0) |

Abbreviations: ICP=intracranial pressure, ICU=intensive care unit, TIL=Therapy Intensity Level scale,^8,9^ TIL^(Basic)^=condensed TIL scale.^8^ The numeric definition of each scale is listed in Table 1, and the calculation of maximum (e.g., TIL_max_) and median (TIL_median_) scores is described in the Methods. The 95% confidence intervals of performance metrics were derived from bootstrapping with 1,000 resamples of unique patients over 100 missing value imputations. Refractory intracranial hypertension was defined as recurrent, sustained (i.e., of at least ten minutes) increases of ICP above 20 mmHg despite medical ICP management during ICU stay. This information was recorded by attending physicians in patient discharge summaries. The rows in bold designate the thresholds which maximise Youden’s J statistic for each scale summary metric.
