# Supplementary material for: Therapy Intensity Level Scale for Traumatic Brain Injury: Clinimetric Assessment on Neuro-Monitored Patients Across 52 European Intensive Care Units
Source: J Neurotrauma. 2024 Apr 4;41(7-8):887–909. doi: 10.1089/neu.2023.0377 (PMC11005383; doi:10.1089/neu.2023.0377)
Supplement: Supplemental data [file Suppl_TableS4.docx]

**Supplementary Table S4. Performance of detecting day of surgical ICP control at each threshold of TIL_24_.**

| **TIL_24_ threshold** (≥) | **Sensitivity** (%) | **Specificity** (%) | **Youden’s J** (%) |
| --- | --- | --- | --- |
| 0 | 100% (100–100%) | 0% (0–0%) | 0% (0–0%) |
| 1 | 100% (100–100%) | 7% (6–7%) | 7% (6–7%) |
| 2 | 100% (100–100%) | 12% (11–13%) | 12% (11–13%) |
| 3 | 100% (100–100%) | 21% (19–22%) | 21% (19–22%) |
| 4 | 100% (100–100%) | 32% (30–33%) | 32% (30–33%) |
| 5 | 100% (100–100%) | 42% (41–44%) | 42% (41–44%) |
| 6 | 97% (95–99%) | 53% (51–55%) | 50% (47–53%) |
| 7 | 95% (93–98%) | 62% (60–64%) | 57% (54–60%) |
| 8 | 93% (90–96%) | 68% (66–70%) | 61% (58–64%) |
| **9** | **87% (83–91%)** | **74% (72–76%)** | **61% (57–65%)** |
| 10 | 79% (75–84%) | 79% (78–81%) | 58% (54–63%) |
| 11 | 72% (67–76%) | 84% (83–86%) | 56% (51–61%) |
| 12 | 66% (61–70%) | 88% (86–89%) | 53% (49–58%) |
| 13 | 57% (53–62%) | 91% (90–92%) | 48% (43–53%) |
| 14 | 50% (45–55%) | 93% (92–94%) | 43% (39–48%) |
| 15 | 43% (39–48%) | 95% (94–96%) | 38% (33–43%) |
| 16 | 36% (32–41%) | 97% (96–97%) | 33% (28–38%) |
| 17 | 30% (26–35%) | 98% (97–98%) | 28% (24–32%) |
| 18 | 24% (20–28%) | 98% (98–99%) | 22% (18–27%) |
| 19 | 20% (16–23%) | 99% (99–99%) | 19% (15–22%) |
| 20 | 15% (12–18%) | 99% (99–100%) | 14% (11–17%) |
| 21 | 12% (9–15%) | 100% (100–100%) | 12% (9–14%) |
| 22 | 10% (8–13%) | 100% (100–100%) | 10% (7–12%) |
| 23 | 8% (6–10%) | 100% (100–100%) | 8% (6–10%) |
| 24 | 6% (4–7%) | 100% (100–100%) | 6% (4–7%) |
| 25 | 4% (2–5%) | 100% (100–100%) | 4% (2–5%) |
| 26 | 2% (1–3%) | 100% (100–100%) | 2% (1–3%) |
| 27 | 1% (1–2%) | 100% (100–100%) | 1% (1–2%) |
| 28 | 1% (0–1%) | 100% (100–100%) | 1% (0–1%) |
| 29 | 1% (0–1%) | 100% (100–100%) | 1% (0–1%) |
| 30 | 1% (0–1%) | 100% (100–100%) | 1% (0–1%) |
| 31 | 0% (0–0%) | 100% (100–100%) | 0% (0–0%) |
| 33 | 0% (0–0%) | 100% (100–100%) | 0% (0–0%) |

Abbreviations: ICP=intracranial pressure, ICU=intensive care unit, TIL=Therapy Intensity Level scale,^8,9^ TIL^(Basic)^=condensed TIL scale.^8^ The numeric definition of TIL is listed in Table 1, and the calculation of daily (e.g., TIL_24_) scores is described in the Methods. The 95% confidence intervals of performance metrics were derived from bootstrapping with 1,000 resamples of unique patients over 100 missing value imputations. If a decompressive craniectomy was performed as a last resort for refractory intracranial hypertension, each of the days following the operation were also considered days of surgical ICP control. The row in bold designates the threshold which maximises Youden’s J statistic.
