# Supplementary material for: Therapy Intensity Level Scale for Traumatic Brain Injury: Clinimetric Assessment on Neuro-Monitored Patients Across 52 European Intensive Care Units
Source: J Neurotrauma. 2024 Apr 4;41(7-8):887–909. doi: 10.1089/neu.2023.0377 (PMC11005383; doi:10.1089/neu.2023.0377)
Supplement: Supplemental data [file Suppl_FigS4.pdf]

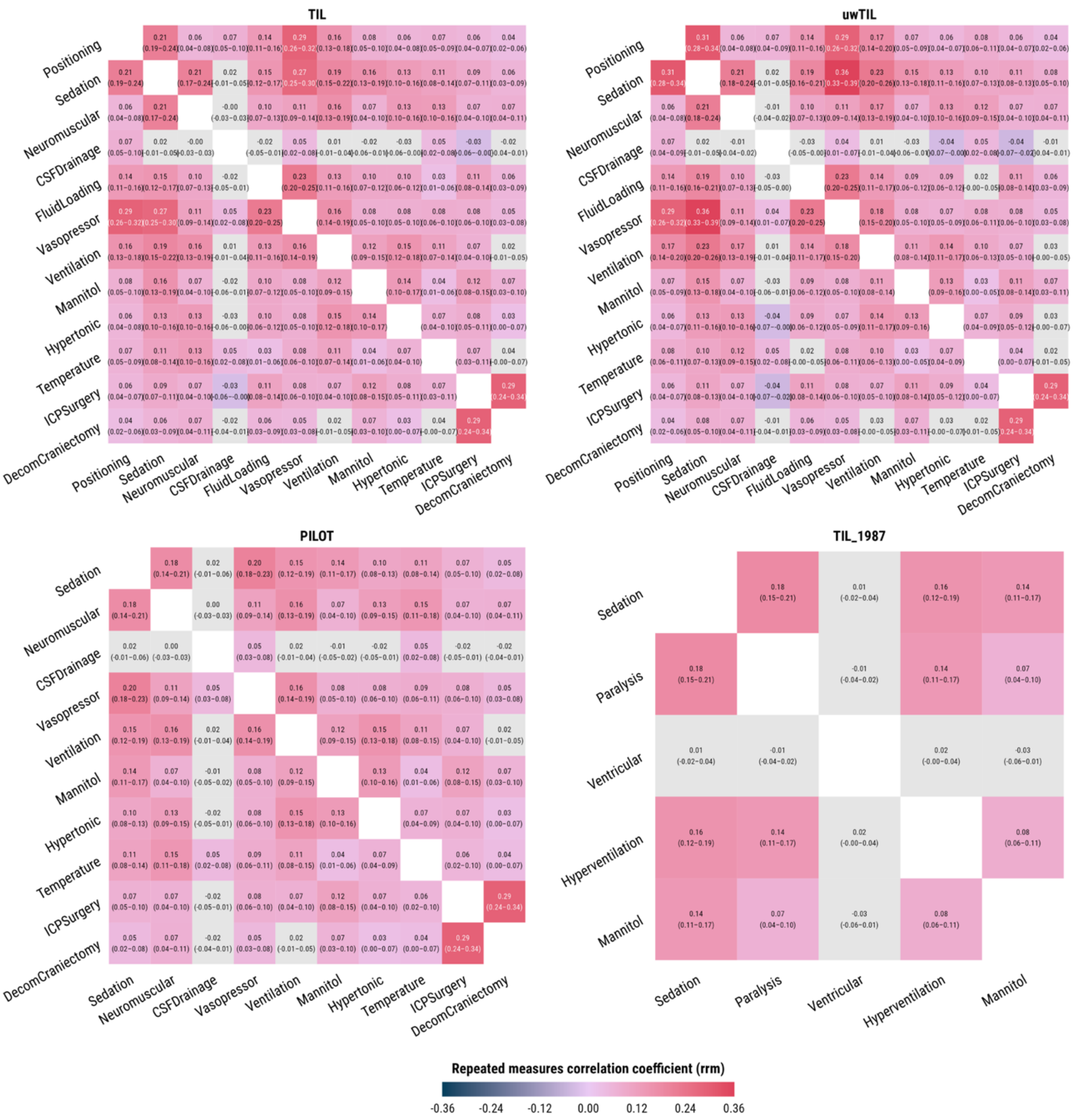

Supplementary Figure S4. Inter-item correlation matrices for daily scores of TIL and alternative scales. Abbreviations: ICU=intensive care unit, PILOT=Paediatric Intensity Level of Therapy scale,[7] TIL=Therapy Intensity Level scale,[8,9] TIL$^{(1987)}$=original Therapy Intensity Level scale published in 1987,[6] TIL$^{(Basic)}$=condensed TIL scale,[8] uwTIL=unweighted TIL scale in which sub-item scores are replaced by the ascending rank index within the item. The items and associated scores of each scale are described in Table 1. The values in parentheses represent 95% confidence intervals derived from bootstrapping with 1,000 resamples of unique patients

over 100 missing value imputations, and light grey boxes designate statistically insignificant correlations.
