# Supplementary material for: Therapy Intensity Level Scale for Traumatic Brain Injury: Clinimetric Assessment on Neuro-Monitored Patients Across 52 European Intensive Care Units
Source: J Neurotrauma. 2024 Apr 4;41(7-8):887–909. doi: 10.1089/neu.2023.0377 (PMC11005383; doi:10.1089/neu.2023.0377)
Supplement: Supplemental data [file Suppl_FigS5.pdf]

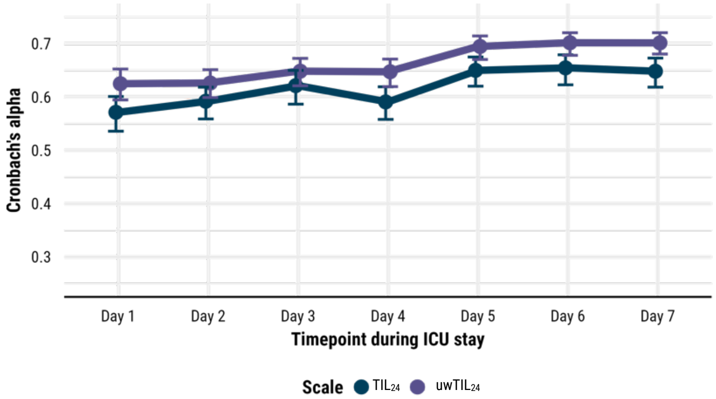

Supplementary Figure S5. Internal reliability of TIL and uwTIL. Abbreviations: ICU=intensive care unit, TIL_{24}=Therapy Intensity Level scale score of calendar day,[8,9] $uwTIL_{24}$=unweighted TIL scale score of calendar day in which sub-item scores are replaced by the ascending rank index within the item. The numeric definition of each scale is listed in Table 1, and the calculation of daily (e.g., TIL_{24}) scores are described in the Methods. The bars represent 95% confidence intervals derived from bootstrapping with 1,000 resamples of unique patients over 100 missing value imputations.
